# Evidence That Ion-Based Signaling Initiating at the Cell Surface Can Potentially Influence Chromatin Dynamics and Chromatin-Bound Proteins in the Nucleus

**DOI:** 10.3389/fpls.2019.01267

**Published:** 2019-10-17

**Authors:** Antonius J.M. Matzke, Wen-Dar Lin, Marjori Matzke

**Affiliations:** Institute of Plant and Microbial Biology, Academia Sinica, Taipei, Taiwan

**Keywords:** genetically encoded voltage indicator, confocal imaging, chromatin dynamics, fluorescence chromosome tagging, ion-based signaling, pH, SEpHluorin

## Abstract

We have developed tools and performed pilot experiments to test the hypothesis that an intracellular ion-based signaling pathway, provoked by an extracellular stimulus acting at the cell surface, can influence interphase chromosome dynamics and chromatin-bound proteins in the nucleus. The experimental system employs chromosome-specific fluorescent tags and the genome-encoded fluorescent pH sensor SEpHluorinA227D, which has been targeted to various intracellular membranes and soluble compartments in root cells of *Arabidopsis thaliana*. We are using this system and three-dimensional live cell imaging to visualize whether fluorescent-tagged interphase chromosome sites undergo changes in constrained motion concurrently with reductions in membrane-associated pH elicited by extracellular ATP, which is known to trigger a cascade of events in plant cells including changes in calcium ion concentrations, pH, and membrane potential. To examine possible effects of the proposed ion-based signaling pathway directly at the chromatin level, we generated a pH-sensitive fluorescent DNA-binding protein that allows pH changes to be monitored at specific genomic sites. Results obtained using these tools support the existence of a rapid, ion-based signaling pathway that initiates at the cell surface and reaches the nucleus to induce alterations in interphase chromatin mobility and the surrounding pH of chromatin-bound proteins. Such a pathway could conceivably act under natural circumstances to allow external stimuli to swiftly influence gene expression by affecting interphase chromosome movement and the structures and/or activities of chromatin-associated proteins.

## Introduction

The development, viability and environmental responsiveness of unicellular and multicellular organisms depends on the integration of diverse molecular, chemical and mechanical signaling pathways that ultimately exert an effect on gene expression. In addition to these classical signaling pathways, electrical signaling is increasingly recognized as an essential and rapid means to transmit information within and between cells and throughout whole organisms (Levin and Stevenson, 2012; Mousavi et al., 2013; Cohen and Venkatachalam, 2014; Choi et al., 2016; Hedrich et al., 2016; Gilroy et al., 2018). On the cellular level, the electrical component at the plasma membrane (PM) comprises both the trans-membrane potential, which is generated primarily by proton gradients in plants (Duby and Boutry, 2009), and the surface potential, which represents the electrical potential over a small distance from the plane of the membrane. Surface potentials are normally sufficiently negative to enrich for cations at the membrane surface (Kinraide et al., 1998; Cohen and Venkatachalam, 2014). Plant endomembranes also contain electrogenic proton pumps, which together with counter ion fluxes establish cell-internal pH gradients that are important for responses of plants to a variety of developmental and environmental cues (Sze and Chanroj, 2018).

We are interested in testing the hypothesis that extracellular stimuli can influence gene expression *via* a rapid electrical/ion-based pathway acting from the cell surface to the nucleus through interconnected intracellular membrane systems (Matzke and Matzke, 1991; Matzke et al., 2010b). Electrical conveyance of information throughout the cell by means of intracellular membranes has been envisaged as a “cytoplasmic nervous system” (Sepheri-Rad et al., 2018) and proposed to feature the endoplasmic reticulum (ER), which is closely associated with the PM through membrane contact sites (Wang et al., 2017) and physically continuous with the outer nuclear membrane (ONM) (Matzke and Matzke, 1991; Sepheri-Rad et al., 2018). Electrical signals could trigger multiple events at membranes including opening of voltage-gated ion channels; changes in surface potentials by release of membrane-bound ions; and alterations in the structures and/or activities of integral membrane proteins, as well as membrane-associated proteins and other polyelectrolytes such as DNA (Matzke and Matzke, 1991; Bezanilla, 2008).

Investigating electrical/ion-based pathways and their effects on the nuclear genome in living cells requires non-invasive techniques for assessing changes in trans-membrane electrical potentials and concentrations of soluble and membrane-associated ions, as well as a means to detect effects at the genomic DNA level. Ideally, these techniques should allow changes to be monitored simultaneously at multiple membrane systems and at chromatin within individual cells, as well as across populations of cells in intact organisms. Genome-encoded fluorescent sensors of membrane voltage and of various ions, such as H^+^ and Ca^+2^, together with methods for fluorescent-tagging of interphase chromosomes and three-dimensional (3D) live cell imaging technology represent useful tools for such studies.

Genome-encoded voltage indicators (GEVIs) have been developed and used in animal systems for the detection of coordinated changes in PM potential in different subpopulations of neuronal cells (Knöpfel, 2012). Different GEVIs are based on either changes in Fo‶rster resonance energy transfer (FRET) or fluorescence intensity (Matzke and Matzke, 2013; Knöpfel et al., 2015). FRET-based GEVIs rely on two differently colored fluorescent proteins, which shift in proximity in response to changes in trans-membrane potential, thus positioning the two proteins to favor the occurrence of FRET. By contrast, fluorescence intensity-based GEVIs comprise only a single fluorescent protein that shows alterations in fluorescence intensity following changes in membrane voltage. Although the former can be used to quantify shifts in membrane potential, currently available intensity-based GEVIs provide a qualitative assessment of membrane voltage changes (Cohen and Venkatachalam, 2014; Hilleary et al., 2018).

GEVIs are beginning to be adapted for use in plant systems. We have tested both FRET- and intensity-based GEVIs in root cells of stably transformed *Arabidopsis* seedlings. In our experiments, several genome-encoded FRET-based GEVIs, under the control of either the *RPS5A* promoter or the constitutive UBI-10 promoter were not expressed strongly enough to detect changes in PM voltage (Matzke and Matzke, 2013). However, a transiently expressed FRET-based GEVI was used successfully to study membrane trafficking involving a SNARE protein in cells of the root elongation zone (Grefen et al., 2015).

We also tested the fluorescence intensity-based GEVI ArcLight, which is a voltage-sensing derivative of the fluorescent pH-sensing protein SEpHluorin (Miesenböck, 2012; Han et al., 2014). The voltage sensitivity of ArcLight was achieved by adding the voltage-sensing domain of *Ciona intestinalis* voltage-sensing phosphatase (Ci-VSD) to SEpHluorin, and changing amino acid 227 from alanine to aspartic acid (A227D) (Han et al., 2014). Arclight has been used in metazoan systems to study changes in PM voltage, which is largely determined in animal cells by the trans-membrane distributions of Na^+^ and K^+^ ions. In plant cells, however, where H^+^ ions are the major contributor to the membrane potential, the pH sensitivity of the SEpHluorin-moiety of ArcLight appears to override its voltage sensitivity (Matzke and Matzke, 2015). The predominance of pH sensing was revealed by the identical responses of ArcLight and SEpHluorinA227D (ArcLight without the Ci-VSD; referred to hereafter as SEpHluorinD) in root cells following application of extracellular ATP (eATP) (Matzke and Matzke, 2015). In animals and plants, eATP functions as an external signaling molecule that triggers a cascade of responses including changes in cytosolic calcium, which are coincident with changes in cytosolic pH (Felle, 2001; Behera et al., 2018), as well as changes in membrane conductance (Tanaka et al., 2010; Cao et al., 2014) and gene expression (Jewell et al., 2019). Therefore, even though ArcLight does not appear to be suitable for directly detecting changes in membrane voltage in plant systems, it can be used as an intensity-based fluorescent sensor of qualitative shifts in pH, which can occur as a downstream consequence of changes in membrane potential. Able to act as either a signal or a messenger in plant cells, pH has important roles in mediating responses to multiple environmental cues and influencing gene expression (Felle, 2001).

Among the techniques available for observing DNA in living cells, fluorescent labeling of specific chromatin sites using bacterial Tet and Lac operator-repressor systems has proven useful in a number of studies on chromatin dynamics in yeast, *Drosophila* and plants (Weiss et al., 2018). This method is based on binding of a fusion protein comprising a bacterial repressor protein (RP) and a fluorescent protein (FP) to the cognate operator repeats introduced as a tandem array into the host genome by transformation procedures. The tagged genomic sites can be visualized as bright fluorescent dots under a fluorescence microscope. We combined the Tet and Lac systems for two-color fluorescence labeling of chromosome sites in *Arabidopsis* and demonstrated considerable variability of 3D interphase chromatin arrangement in root cells (Matzke et al., 2003; Matzke et al., 2005; Matzke et al., 2008; Matzke et al., 2010). Use of the Lac system to study chromatin dynamics in yeast and *Drosophila* indicated that interphase chromatin is not static but undergoes significant, constrained diffusive motion (“jiggling”) within the nucleus, such that a given chromatin segment oscillates within a restricted nuclear sub-region with a typical radius of 0.5 to 0.7 μm (Marshall et al., 1997; Soutoglou and Misteli, 2007; McNally, 2009; Bronshtein et al., 2016). Similar observations using the Lac system were made in plants (Kato and Lam, 2003; Rosin et al., 2008). Local diffusional motion of chromatin has been suggested to be important for gene regulation, possibly because it permits a locus to enter nuclear environments favorable for optimal expression (Soutoglou and Misteli, 2007).

Using the tools described above, we are testing the aforementioned hypothesis of an electrical/ion-based signaling pathway operating from the PM to the nucleus to elicit changes at the genome level. In a partial test of the hypothesis, we previously used membrane-targeted SEpHluorinD to demonstrate that eATP provokes virtually instantaneous and synchronous reductions in pH at the PM and the inner nuclear membrane (INM) of root cells (Matzke and Matzke, 2015). This finding is consistent with rapid inter-membrane communication that affects proton activities at both membrane surfaces. Here we describe improved tools and further tests of the hypothesis to investigate possible direct effects of the proposed electrical/ion-based signaling pathway on the genome. Results from pilot studies presented here suggest that eATP can trigger changes in interphase chromatin mobility and the surrounding pH at specific genomic sites concomitantly with eATP-induced changes in pH at the PM and INM.

## Materials and Methods

### Constructs and Plant Material

The building blocks of the constructs and the final constructs used in this study are shown in supplementary **Data Sheet 1**. SEpHluorinD was targeted to the PM using a CBL1 motif and to the INM and ONM using SUN2 and WPP motifs, respectively (Matzke and Matzke, 2015). Depending on whether the SUN2 motif is positioned at the N- or C-terminus, SEpHluorinD can be on the nucleoplasmic face of the INM or within the perinuclear space (compartment between INM and ONM). We found that the latter variant gives a cleaner signal (**Data Sheet 2** in supplement) and therefore this version of SUN2-SEpHluorinD was used to produce the results presented in this paper.

Because fluorescence of SEpHluorin is highest around pH 7.5 and extinguished at pH 5.5 (Miesenböck, 2012), it is suitable for qualitative assessments of shifts in pH within this range. A decrease in pH results in a reduction of the fluorescence intensity of SEpHluorin with a maximum visible change of approximately 2 pH units (Miesenböck, 2012). SEpHluorinD tethered to different membranes using specific targeting motifs as described above provides an indication of proton activity at the membrane surface. The rationale for the estimated reductions in pH reported in this paper following eATP treatment is shown in supplementary **Data Sheet 3**.

The chromosomal positions of the T-DNA insertions containing either *lac* or *tet* operator repeats and a gene encoding the cognate repressor protein (RP)-fluorescence protein (FP) fusion proteins (TetR-YFP and either LacR-dsRed2 or EGFP) are depicted in supplementary **Data Sheet 4**. The genes encoding the fusion proteins, which are under the transcriptional control of the 35S promoter, have become silenced over time (Matzke et al., 2010b). Therefore, for the present study, a second T-DNA encoding the desired RP-FP fusion protein under the transcriptional control of the *RPS5A* promoter (At3g11940) (Weijers et al., 2001), was introduced (**Data Sheet 1** in supplement). Because lines 16:101, 16:112 and 5:106 display the strongest and most reliable fluorescent dots, possibly owing to large, complex insertions of the operator repeats (Matzke et al., 2008), they have been used to generate the results shown in this report.

The Arabidopsis (*Arabidopsis thaliana*) transgenic lines used in this study are in the Col-0 ecotype (Matzke et al., 2005). Transgenic lines for which data are reported here did not show obvious developmental defects when cultivated under standard conditions (see below), and after data collection at the seedling stage, could be planted in soil for further growth and seed set. Seeds of the 16 operator repeat-containing Arabidopsis lines are available from the Arabidopsis Biological Resource Center (Ohio University, USA) in selected double insertions under the stock numbers CS72296-CS72301 and in single insertions under stock numbers CS72302-CS72317.

### Fixation of Plant Seedlings

Seedlings were fixed for 5 min in ice-cold methanol and 30 s in ice-cold acetone. The seedlings were immediately dried between paper towels and then transferred to distilled water until mounting on a slide in imaging buffer for confocal microscopy (Kato and Lam, 2003).

### Confocal Microscopy and Acquisition of Data to Analyze Fluorescent Intensity Changes of Membranes and Alleles, and Chromatin Dynamics

Confocal microscopy (using a Leica TCS LSI confocal microscope equipped with a 63× oil immersion objective) was used to acquire 3D time-lapse data. We routinely image the area of the transition zone because the nuclei in this region are mostly round and non-mobile, features that improve the ability to study chromatin dynamics over time and after eATP addition.

#### Growth and Mounting of Seedlings for Confocal Microscopy and Addition of eATP During Acquisition

Arabidopsis seedlings were grown vertically in square petri dishes containing solid Murashige and Skoog (MS) medium in an incubator at 22°C to 25°C under a 16-h light/8-h dark cycle. When the roots were approximately 2.5 cm in length, the seedlings were mounted in a sterile hood on 25 × 75 × 1.0 mm microscope slides (SUPERFROST PLUS, Thermo Scientific Art. No. J1800AMNZ) in 63-µl imaging solution [5-mM potassium chloride, 10-mM MES hydrate, 10-mM calcium chloride, adjusted to pH 5.8 with Tris(hydroxymethyl)aminoethane) (Loro et al., 2012)]. The root was then covered with a 24 × 40-mm microscope cover glass Nr.1 (Marienfeld laboratory glassware Ref. 0101192) in which a perforation of approximately 1.5 mm in diameter (centered 3 cm away from the right edge of the slide) had been drilled under water with a diamond coated 1.5-mm drill (Kralj et al., 2011). The root tip was positioned approximately 5 mm from the perforation and the cover slip was sealed at the edges with rubber cement (Fixogum Art.-Nr. 2901 10 000, Marabu, Germany). The microscope slide was placed into the slide holder of a Leica TCS LSI confocal microscope equipped with a 63× oil immersion objective (**Data Sheet 5** in supplement).

After adding a drop of TypeF Immersion liquid (Leica Cat. Nr. 11 513 859) on the coverslip approximately one centimeter above the root tip, the objective was lowered until it touched the immersion liquid. Under visual inspection through the oculars, the root was observed and followed until the transition zone of the root tip was centered in the viewing field. Live mode imaging was used to adjust intensity and gain settings of the lasers 488 nm for the green channel and 532 nm for the red channel. For eATP experiments, acquisition was set to 31 min with acquisition of 21 pictures in 1 µm distances every minute first for the green channel and second for the red channel.

For eATP addition, 7 µl of a freshly made ATP solution (100-mM adenosine 5´-triphosphate disodium salt hydrate, Sigma A2383 in imaging solution adjusted to pH 5.8 with Tris(hydroxymethyl)aminomethane, Merck 1.08382) was pipetted with a Gilson P20 into Teflon tubing mounted through a blue pipette tip on a holder on the microscope stage and positioned right over the perforation in the cover slip (**Data Sheet 5** in supplement). We found that the best distance of root tip to the perforation in the cover slip is approximately 5 mm. The leaves of the seedling were then covered with a Parafilm “globe” (produced by stretching the Parafilm in a small area with a thumb) to prevent desiccation during the acquisition period. Imaging was started and during the thirteenth red channel acquisition, the 7-µl 100-mM ATP solution was pipetted into the perforation of the cover slip. The 100 mM ATP added at the perforation site on the coverslip is likely to become diluted to a lower concentration during diffusion to the root tip (distance ca. 0.5 cm) in the thin layer of imaging buffer. After the acquisition period the seedling was removed from the slide and put on an MS agar plate for recovery overnight. Seedlings remain viable and can be planted in soil for seed harvest.

### Confocal Data Analysis

**Figure 1:**
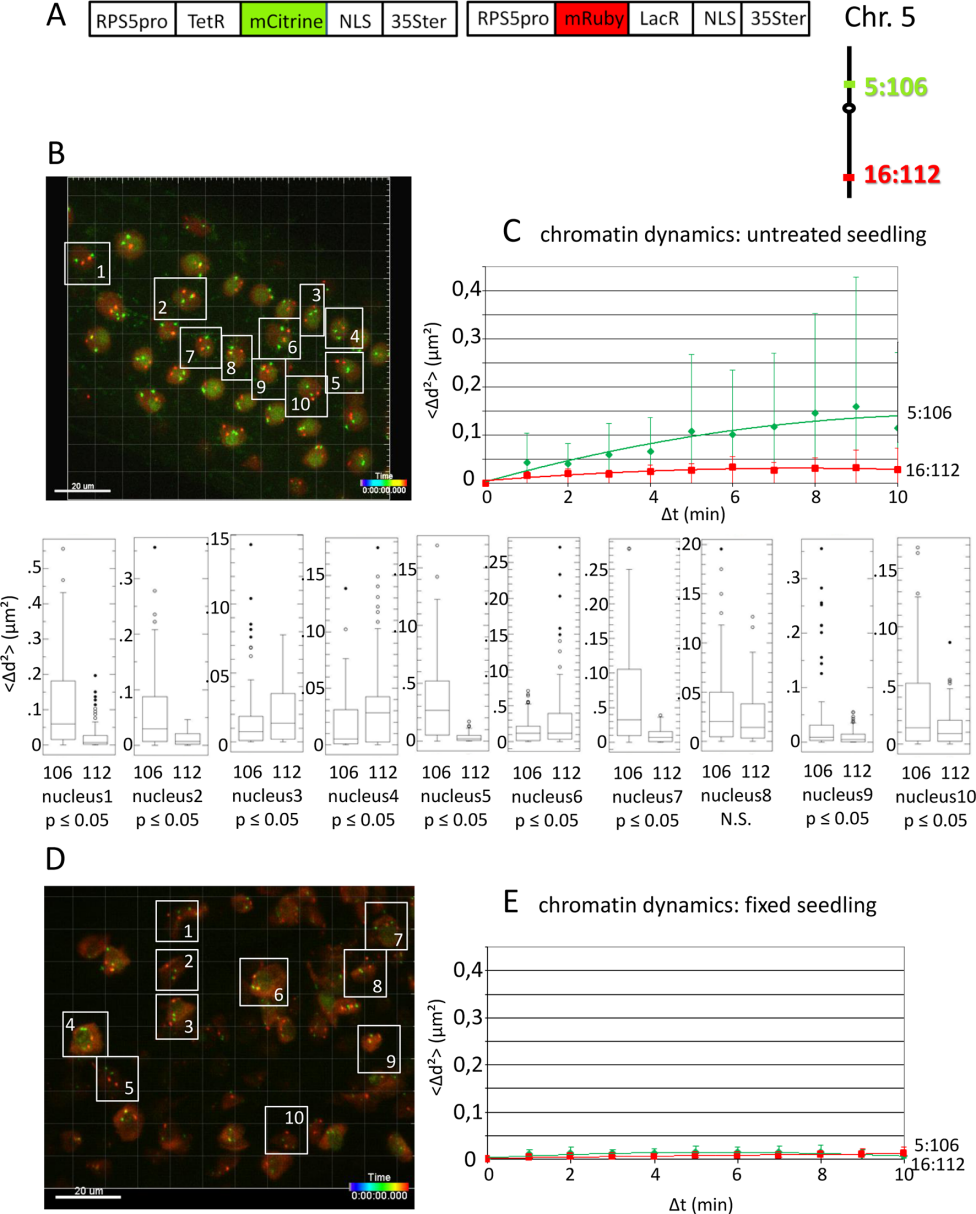
Analysis of chromatin dynamics in untreated plants. **(A)** The construct encoded two nuclear-localized fusion proteins: TetR-mCitrine and mRuby-LacR (**Data Sheet 1**, combination **Figure 1**, in supplement). Both genes are under the transcriptional control of the *RPS5A* promoter (RPS5pro) and the 35S terminator (35Ster). NLS, nuclear localization signal. Right: TetR-mCitrine and mRuby-LacR fusion proteins bind, respectively, to *tetO* repeats at locus 5:106 and *lacO* repeats at locus 16:112. The two loci are integrated on the top and bottom arms of chromosome 5, respectively (green, locus 5:106; red, locus 16:112). **(B)** Left: Confocal image (maximum projection; enlargement in **Data Sheet 9** in supplement) at time point t1 of fluorescent-tagged loci 5:106 and 16:112 in nuclei of cells in the root transition zone. Two red and two green dots are visible in most nuclei. Nuclei boxed in white were used to measure distances between the two red alleles and the two green alleles in the same nucleus. For confocal microscopy, *Arabidopsis* seedlings harboring these fluorescent-tagged loci were mounted on a slide in imaging buffer. One 3D data set, allowing measurement of allelic distances in Imaris, was acquired from both the red and green channels every minute (21 planes) over a time period of 10 min (**Video 3**). A total of 10 data records (one for each nucleus), each containing eleven time points, was analyzed (**Table 2**, **sheets 1**–**2**, in supplement). **(C)** Chromatin dynamics in living cells was determined by plotting the cumulative overall mean squared change in distance between the two alleles <Δd^2^>(μm^2^) against elapsed time intervals Δt. The plateau height of the trendline reflects the size of the confinement region. Higher plateau values indicate increased chromatin movement (Qian et al., 1991). The graph shows a scatterplot of the <Δd^2^> values (cumulated (squared) distance travel since t0) for 10 nuclei over a period of 10 min (Δt = elapsed time since t0) and is overlaid with order two polynomial trendlines and standard deviation bars are shown. The Δd^2^(μm^2^) rises to 0.14 for locus 5:106 and 0.03 for locus 16:112 during the 10-min data acquisition period in this experiment. These Δd^2^(μm^2^) values correspond to radiuses of confinement of 0.4 μm and 0.17 μm, respectively, in nuclei with an approximate diameter of 10 μm, which is in line with previous results (Marshall et al., 1997; Kato and Lam, 2003). Single nuclei box plot analyses and calculation of *p* values using the Δd^2^ values revealed nine nuclei with significantly different changes in chromatin mobility (*p* ≤ 0.05) between locus 5:106 and locus 16:112 (**Table 3**). N.S., not significant. **(D)** Same procedure as in part **C** using fixed seedlings as a negative control for “jiggling” of alleles. Left: nuclei boxed in white used for measurements (**Table 2**, **sheets 3**–**4** in supplement). **(E)** Chromatin dynamics (fixed seedling): <Δd^2^>(μm^2^) of fixed seedlings against elapsed time intervals (**Table 4**, in supplement). Motion in living cells (part **C**) is greater than in fixed cells, indicating that the movement is not due to measurement error.

**Figure 2:**
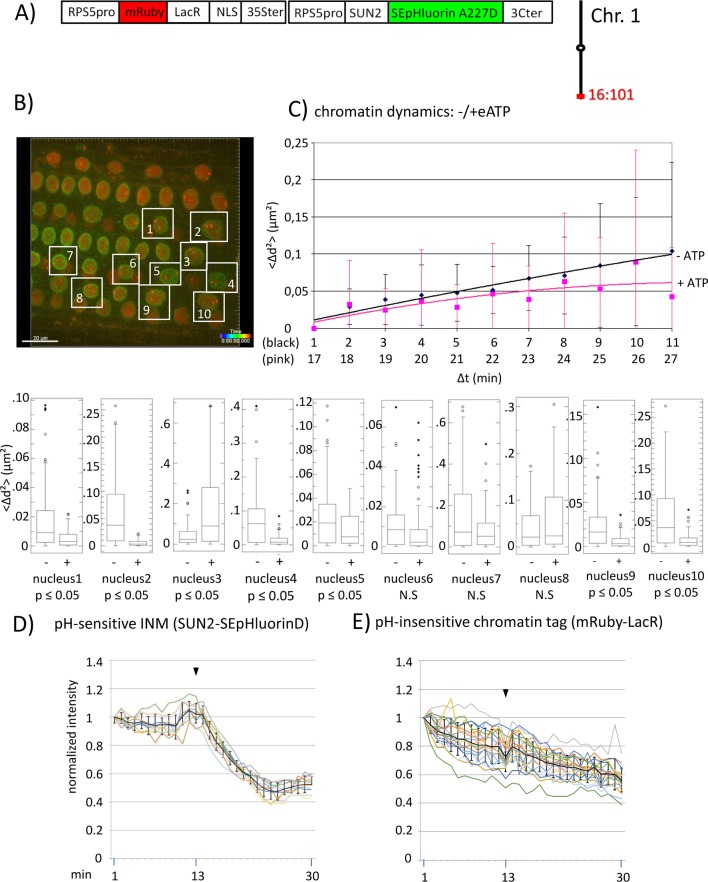
Chromatin dynamics and fluorescence intensity changes at the INM following addition of eATP. **(A)** The construct encoded nuclear-localized mRuby-LacR fusion protein (pH-insensitive) and, in opposite orientation, SUN2-SEpHluorin227D, which is pH-sensitive and localized to the perinuclear face of the INM *via* the SUN2 targeting signal. Both genes are under the transcriptional control of the RPS5pro; the first contains the 35ter and the second contains the 3C terminator (3Cter). For readability, the module encoding RPS5pro-SUN2-SEpHluorin-3C is in the orientation shown but it is actually in the opposite orientation (**Data Sheet 1**, combination **Figure 2**, in supplement). (**Data Sheet 1**, combination **Figure 2**, in supplement). Right: the mRuby-LacR fusion protein binds to *lacO* repeats integrated at locus 16:101 on the bottom arm of chromosome 1. **(B)** Confocal image (maximum projection; enlargement in supplementary **Data Sheet 9**) at time point t1 of fluorescent-tagged locus 16:101 in nuclei of cells in the root transition zone. Two red dots are visible in most nuclei. Nuclei boxed in white were used to measure distances between the two red alleles and fluorescent intensity analysis (parts **D** and **E**). **(C)** Chromatin dynamics: *Arabidopsis* seedlings harboring the above construct were mounted on a slide in imaging buffer for confocal microscopy. A 3D data set allowing measurement of allelic distances in Imaris was acquired every minute (21 planes) over a time period of 30 min during which eATP was added at 13 to 14 min during red channel acquisition. A total of 10 data records (10 nuclei), each containing 30 time points, was collected (**Table 2**, **sheet 5** in supplement). Chromatin dynamics was determined as described in the legend to **Figure 1**. The graph shows a scatterplot of the <Δd^2^> values [cumulative (squared) distance travel since t0 and after eATP addition since t17], and standard deviation bars (**Table 5** in supplement) for 10 nuclei over a period of 30 min (Δt = elapsed time since t0, and after eATP addition since t17) and is overlaid with order 2 polynomial trendlines, which indicate “jiggling” before (black) and after (pink) eATP addition. To detect differences in mobility of locus 16:101 before and after eATP treatment, the analysis was restarted following the addition of eATP (following cessation of dislocation turbulence), hence producing two lines. Time points 12-16 were excluded from the analysis owing to data acquired during dislocation turbulence caused by addition of eATP. Single nuclei box plot analyses and calculation of *p* values using the Δd^2^ values revealed seven nuclei with significantly different changes in mobility (*p* ≤ 0.05) of locus 16:101 following eATP treament. N.S., not significant; **(D)** Normalized fluorescence intensity values of all 10 white-boxed nuclei shown in part **B** are overlaid in one graph together with calculated average values, to which standard deviation error bars were added (normalized and non-normalized numbers can be viewed, respectively, in **sheets 1** of **Tables 1** and **10** in supplement). Arrowheads indicate addition of eATP at time point 13. Using these normalized data, the calculated difference in the magnitude of the drop in fluorescence intensity in SUN2-SEpHluorinD versus bleaching of mRuby-LacR between time points 14-25 in response to eATP is statistically significant (*p* ≤ 0.05). In supplementary **Data Sheet 8**, part **Figure 2D**, ΔpH values are shown under each nucleus1-10 [maximum ΔpH 0.9 (nucleus 7), minimum ΔpH 0.3 (nucleus 2); average (n = 10) 0.6]. Fluorescence intensity at INM is read in spot objects (spot size approximately 10 μm) capturing punctual fluorescence. **(E)** Response of the pH-insensitive mRuby-LacR chromatin tag following eATP treatment: normalized intensities of all 10 nuclei overlaid in one graph (numbers before normalization can be viewed in supplementary **Table 1**, **sheet 2**). Fluorescence intensities of individual nuclei are shown in supplementary **Data Sheet 8**, part **Figure 2E**.

**Figure 3:**
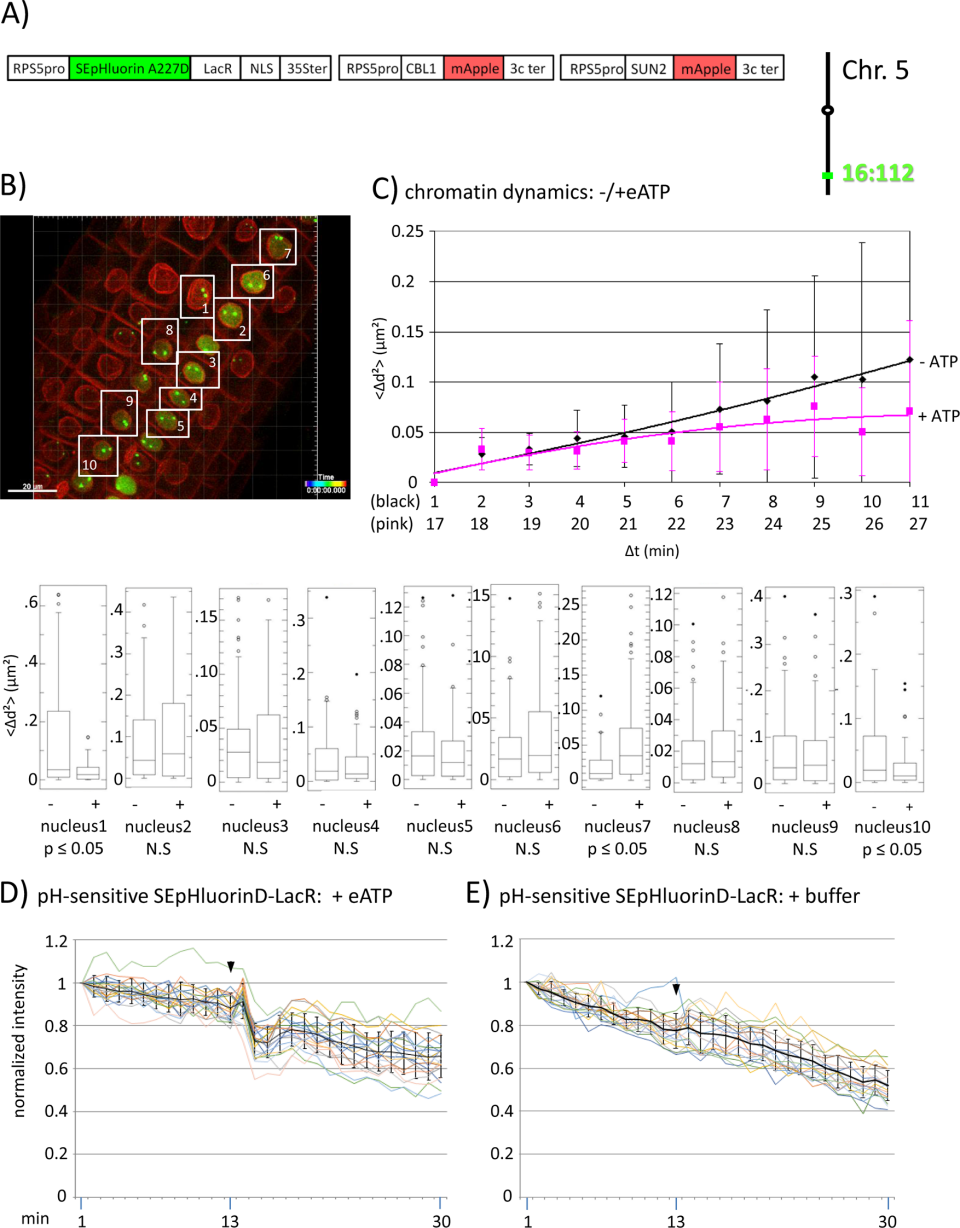
Fluorescence intensity changes of a pH-sensitive fluorescent DNA-binding protein following addition of eATP. **(A)** The construct encoded nuclear-localized SEpHluorinD-LacR fusion protein as a pH-sensitive fluorescent DNA-binding protein, and SUN2-mApple and CBL1-mApple as INM and PM visual markers. All three genes are under the transcriptional control of the RPS5 promoter; the first contains the 35ter and the last two contain the 3C terminator (3Cter) for readability, the module encoding RPS5pro-LacR is in the orientation shown but it is actually in the opposite orientation (**Data Sheet 1**, combination **Figure 3**, in supplement). Right: The SEpHluorinD-LacR fusion protein binds to *lacO* repeats integrated at locus 16:112 on the bottom arm of chromosome 5. **(B)** Confocal image (maximum projection; enlargement in **Data Sheet 9** in supplement) at time point t1 of homozygous fluorescent-tagged locus 16:112 in nuclei of cells in the root transition zone. Two green dots are visible in most nuclei. Nuclei used for chromatin dynamics analyses (part **C**) and fluorescence intensity analysis (parts **D** and **E**) are boxed in white. **(C)** Chromatin dynamics: Mounting, confocal microscopy, eATP treatment, data acquisition and data analysis of *Arabidopsis* seedlings harboring the above construct were carried out as described in the legend to **Figure 2**. Chromatin dynamics was determined as described in the legend to **Figure 1**. The graph shows a scatterplot of the average Δd^2^ (<Δd^2^>) values [cumulative (squared) distance travel since t0 and after eATP addition since t17], and standard deviation bars (**Table 2**, **sheet 6** in supplement) for 10 nuclei over a period of 30 min (Δt = elapsed time since t0) and is overlaid with order 2 polynomial trendlines, which indicate “jiggling” before (black) and after (pink) eATP addition. Time points 12-16 were excluded from the analysis owing to unreliable data acquired during dislocation turbulence caused by addition of eATP. To detect differences in the mobility of locus 16:112 before and after eATP treatment, the analysis was restarted following the addition of eATP, hence producing two lines. Bottom: Single nuclei box plot analyses and calculation of *p* values using the Δd^2^ values revealed three nuclei with significantly different changes (*p* ≤ 0.05) in the mobility of locus 16:112 following eATP treatment. N.S., not significant. **(D)** pH-sensitive SEpHluorin-LacR chromatin tag: normalized fluorescence intensity profiles of the two 16:112 alleles in the 10 white-boxed nuclei (numbered in white in part **B**) are overlaid in one graph together with the calculated average values, to which standard deviation bars were added. eATP addition is indicated with a black arrowhead at frames 13-14. Fluorescence intensities of individual nuclei are shown in supplementary **Data Sheet 8**, part **3D**, in which the boxed areas in the fluorescent intensity graphs highlight the region of interest. ΔpH values are shown under each nucleus1-10 for both alleles [maximum ΔpH 0.4 (nucleus 9); minimum ΔpH 0.1 (nuclei 4 and 5); average (n = 20) 0.2]. Normalized and non-normalized data are shown, respectively, in **Data Sheets 3** of **Tables 10** and **1**. Using the normalized data, the calculated difference in the magnitude of the drop in fluorescence intensity in SEpHluorinD-LacR plus eATP versus bleaching in the buffer control between the time points 14-16 points is statistically significant (*p* ≤ 0.05). **(E)** pH-sensitive SEpHluorinD-LacR chromatin tag: Buffer control without eATP (original data can be viewed in supplementary **Table 1**, **sheet 4**). The spikes in fluorescence reflect dislocation turbulence, which occurs upon addition of eATP or buffer. The fluorescence intensity at the genomic location is read in spots objects (1-2.8 μm) capturing punctual fluorescence of the tagged regions.

**Figure 4:**
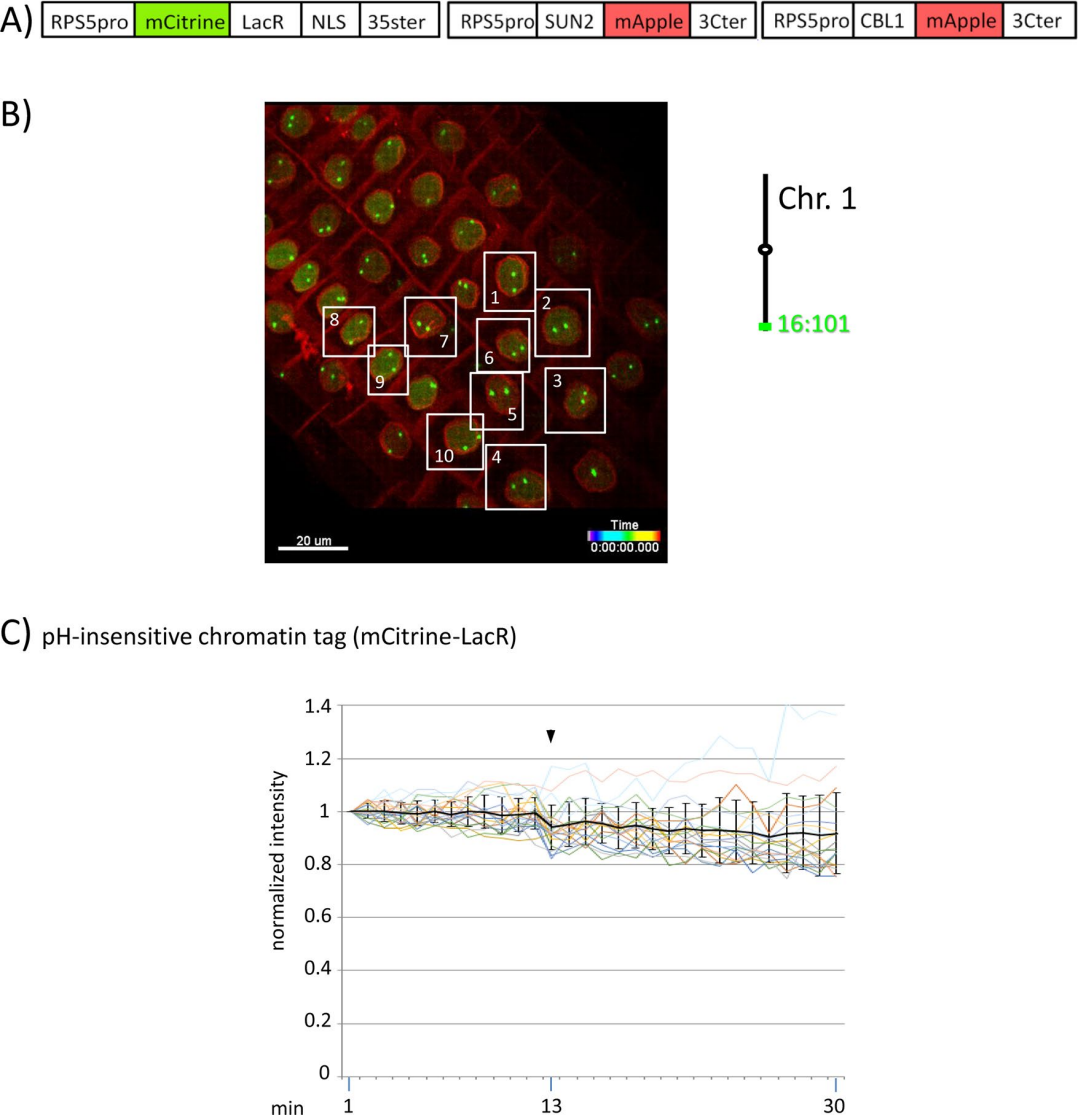
Fluorescence intensity of a pH-insensitive fluorescent DNA-binding protein (mCitrine-LacR) following addition of eATP. **(A)** The construct encoded nuclear-localized mCitrine-LacR fusion protein as a pH-insensitive fluorescent DNA-binding protein, and SUN2-mApple and CBL1-mApple as INM and PM visual markers. All three genes are under the transcriptional control of the RPS5 promoter; the first contains the 35ter and the last two contain the 3C terminator (3Cter). For readability, the module encoding RPS5pro-mCitrine-LacR is in the orientation shown but it is actually in the opposite orientation (**Data Sheet 1**, combination **Figure 3**, in supplement). (**Data Sheet 1**, combination **Figure 4**, in supplement). **(B)** Confocal image (maximum projection; enlargement in **Data Sheet 9** in supplement) at time point t1 of homozygous fluorescent-tagged locus 16:101 in nuclei of cells in the root transition zone. Two green dots are visible in most nuclei. Nuclei used for fluorescence intensity analysis (part **C**) are boxed in white. Right: The mCitrine-LacR fusion protein binds to *lacO* repeats integrated at locus 16:101 on the bottom arm of chromosome 1. **(C)** pH-insensitive chromatin tag (mCitrine-LacR): Fluorescence intensity profiles of normalized intensities of all 10 white-boxed nuclei (in part **B**) are overlaid in one graph together with the calculated average values, to which standard deviation bars were added. Fluorescence intensities of individual nuclei can be viewed in supplementary **Data Sheet 8**, part **Figure 4C**. Addition of eATP is indicated with black arrowheads at frames 13-14. Normalized and non-normalized numbers are shown, respectively, in **sheets 5** of **Tables 10** and **1**, in supplement. Using the normalized data, the calculated difference in the magnitude of the drop in fluorescence intensity of SEpHluorinD-LacR (**Figure 3D**) versus bleaching of mCitrine-LacR (this figure) between the time points 14-16 points in response to eATP is statistically significant (*p* ≤ 0.05). Fluorescence intensity at genomic location is read in spot objects (1-2.8 μm) capturing punctual fluorescence.

**Figure 5:**
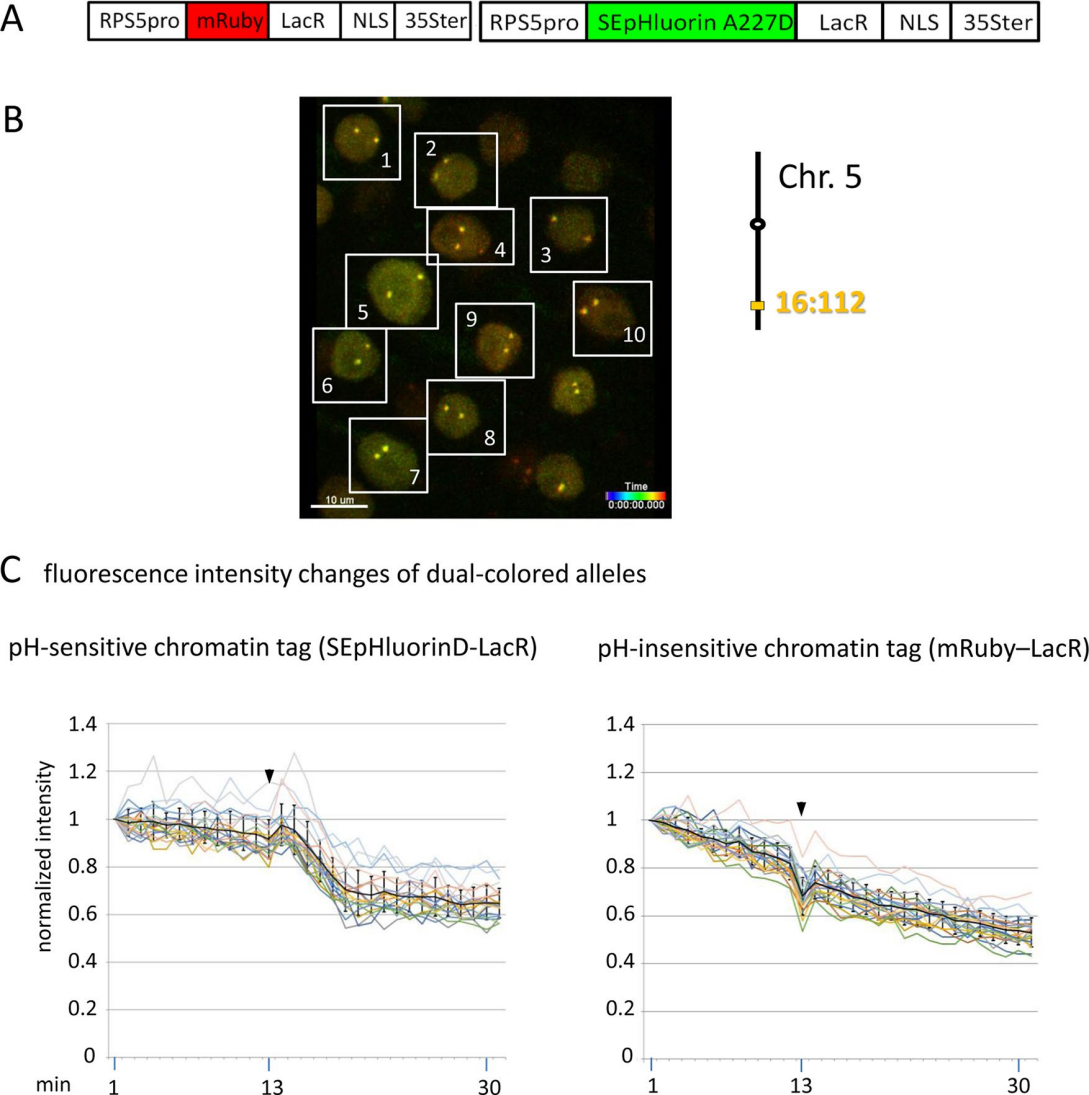
Comparison of fluorescence intensity changes of pH-sensitive and pH-insensitive fluorescent DNA-binding proteins following addition of eATP. **(A)** The construct used in this experiment encoded a nuclear-localized SEpHluorinD-LacR fusion protein as a pH-sensitive fluorescent DNA-binding protein, and mRuby-LacR as a pH-insensitive DNA-binding protein. Both genes are under the transcriptional control of the RPS5 promoter and the 35ter (**Data Sheet 1**, combination **Figure 5**, in supplement). Right: The SEpHluorinD-LacR fusion protein and the mRuby-LacR fusion protein both bind to the tandem *lacO* repeats integrated at locus 16:112 on the bottom arm of chromosome 5. **(B)** Confocal image (maximum projection; enlargement in **Data Sheet 9** in supplement) of double fluorescent-tagged locus 16:112 at time point t1 in nuclei of cells in the root transition zone. Two yellow dots are visible in most nuclei. Nuclei used for fluorescence intensity analysis (part **C**) are boxed in white. **(C)** Fluorescence intensity changes of dual-colored alleles. Response of pH-sensitive tag SEpHluorinD-LacR (left) and pH-insensitive mRuby-LacR (right) to eATP addition (black arrowheads, frames 13-14). Fluorescence intensity profiles of normalized intensities of all 10 nuclei recorded in the green and red channels in the same experiment are overlaid in two separate graphs together with the calculated average values, to which standard deviation bars were added (**Table 10**, **Data Sheet 6**, in supplement). Normalized and non-normalized data can be viewed respectively, in **Data Sheets 6** of **Tables 10** and **1**. Using the normalized data, the calculated difference in the magnitude of the drop in fluorescence intensity of SEpHluorinD-LacR versus mRuby-LacR between the time points 14 to 21 in response to eATP is statistically significant (*p* ≤ 0.05). The spike in fluorescence of the mRuby pH-insensitive tag reflects dislocation turbulence following eATP application. Fluorescence intensities of individual nuclei are shown in supplementary **Data Sheet 8**, part **Figure 5C**. ΔpH values are shown under each nucleus1-10 for both alleles [maximum ΔpH 0.5 (nucleus 5, one allele); minimum ΔpH 0.2 (nuclei 1, 2, 4, 6,9, one allele, nucleus 10 both alleles); average ΔpH 0.3 (n = 20)]. Fluorescence intensity at genomic location is read in spot objects (1–2.8 μm) capturing punctual fluorescence.

The time-lapse data obtained from the experiments described above and presented in **Figures 1**–**5** and Supplementary **Data Sheet 6** were analyzed in Imaris 64 bit software version 7.7.0 (www.bitplane.com). The data were analyzed as described below to determine: fluorescence intensities of INM membrane tagged with SUN2-SEpHluorinD and fluorescence-tagged genomic sites; 3D distances between fluorescent dots/alleles for chromatin dynamics; 3D distances from each allele to the INM; and measurements of nuclear volume.

#### Fluorescence Intensities

Nuclei were isolated using the Crop 3D function resulting in “single nuclei 3D time lapse” Imaris files. These files were used to read the intensity values displayed of detected dots (or “spots”; term used by Imaris software), which represented either fluorescent nuclei with a diameter of approximately 10 µm or fluorescent tagged genomic locations with a diameter of approximately 1 to 2.28 µm (ellipsoid 2-4,56 µm). Intensity values of “spots” were listed in Excel files (**Table 1** in supplement) and graphically displayed in **Figures 2**, **3**, **4** and **5**. Fluorescent intensities were normalized with the start set at 1. Fluorescence intensity data from 10 nuclei were compiled into one graph and supplemented with standard deviation bars. Graphs from individual nuclei before normalization are shown in supplementary **Data Sheet 8**.

#### Estimation of ΔpH

Changes in pH (ΔpH) at all cellular locations and genomic sites was estimated based on the magnitude of reduction of SEpHluorin fluorescence within its known pH range of fluorescence (Miesenböck, 2012) as described in supplementary **Data Sheet 3**. Estimated values of ΔpH were added to graphs of individual nuclei in supplementary **Data Sheet 8**.

#### Patterns in Reductions of Fluorescence Intensity

Graphs showing changes in pH over time at the INM (**Figure 2**), and at three genomic loci (**Figures 2**, **3**, **4** and **5**) show potentially three types of fluorescence change that can occur during the data collection period and affect the pattern of the trace: 1) bleaching, which manifests as a continuous and irreversible decline in fluorescence [most obvious in buffer control (**Figure 3**) and pH-insensitive fluorescent proteins (**Figures 2**, **4**, and **5**)]; 2) dislocation turbulence, which is sometimes observed as sharp spike upon eATP addition through the perforation in the cover slip at time-point 13 (for examples, see supplementary **Data Sheet 8**, nucleus 2 in **Figure 2**, bottom; and **Videos 1** and **2**, time-point 13); and 3) reductions in fluorescence owing to a pH change at the locus under study. The last change is observed as a drop in fluorescence occurring over approximately 2 to 7 min, followed usually by at least partial recovery (most visible in blue boxed nuclei in parts labeled “**Figure 3C**” and “**Figure 5C**” in **Data Sheet 8**, in supplement).

#### Analysis of Chromatin Dynamics

Starting from the “single nuclei 3D time lapse” Imaris files; see above), each time point was isolated using the Crop time function (producing “single nuclei 3D” Imaris files). These files were used to determine the 3D distances between the two dots/alleles of fluorescent tagged genomic locations. After Imaris detection of fluorescent dots/alleles, the dots were separated using the “split” function of Imaris and the distance between them computed using the “compute distances between spots” function. Distance values displayed were entered into Excel files (**Table 2** in supplement). For chromatin dynamic analysis, allelic distances were pasted into an Excel chromatin dynamics analyses file obtained from the Ton Bisseling lab (Laboratory of Molecular Biology, Wageningen University) and modified by Ulf Naumann (Gregor Mendel Institute, Vienna, Austria). The modified Excel file, formatted for data from 10 nuclei and eleven time points (numbers can be adjusted as needed) is available in the Supplementary material as “chromatin dynamics analyses file” (**Table 15** in supplement) which contains 20 sheets (10 nuclei, before and after eATP) plus an “average” sheet. In the chromatin dynamics analysis file, changes in allelic distance (d) during the 1-min time intervals were used to calculate the mean squared distance change in d(t) as <Δ d²> = < [d(t)-d(t+Δ t)]²> and a plot of cumulative traveling distances <Δd^2^(μm^2^)> (cumulated (squared) distance travel since t0) against elapsed time intervals, Δt (delta t = elapsed time since t0), is generated (see **Figures 1**–**3** and supplementary **Data Sheet 6**). The generated curve was replaced by order2 polynomial trendlines using the Excel chart function to visualize plateauing of the values. The height of the plateau of the trendlines reflects the size of the confinement region. The radius of confinement in μm is the square root of that value. Higher plateau values indicate a larger region of confinement, meaning motion (“jiggling”) is less constrained (Marshall et al., 1997; Berg, 2016).

#### Statistical Analyses

For average chromatin dynamics data (**Figures 1C and D**, **Figure 2C** and **Figure3C** and Supplementary **Data Sheet 6C**), standard deviations were calculated in Excel using the STDEV.S function with the Δd² averages from all 10 nuclei at all 10 time points in a given experiment and shown in the figures (**Tables 3**–**7**, column C, lines 27–38, in supplement). Box plot analyses of Δd^2^ values for individual nuclei and calculations of *p* values were performed using the “Data Entry: Student’s t-test” (http://www.physics.csbsju.edu/stats/t-test_bulk_form.html). The Δd^2^ values for individual nuclei were also compared using one-sided KS tests, which confirmed the statistical significance determined by the t-test (**Table 11**_KS_Fig1C, **Table 12**_KS_Fig2C, **Table 13**_KS_Fig3C, **Table 14**_KS_**Data Sheet 6C**, in the supplement; each sheet of these files corresponds to a single nucleus). The *t*-test *p* values are two-sided; a significant *t*-test *p* value means the first set is greater than the second one or vice versa. The KS test used is one-sided; it gives a significant *p* value only when the first set is greater than the second one.

For fluorescence intensity experiments (**Figures 2D and E**, **Figure 3D and E**, **Figure 4C**, and **Figure 5C**), standard deviations of the average fluorescence intensity values of 10 nuclei (boxed in white in the confocal images shown) at each time point were calculated using the STDEV.S function of Excel and added as error bars. To calculate *p* values, the “t test” function of Excel was used to compare normalized fluorescence intensity data (i.e. differences in the drop in fluorescence intensity—high point to low point—during time points indicated in the figures) from pH-sensitive versus pH-insensitive fluorescent proteins or buffer control (**Table 10**, **Data Sheet 7** in supplement). The *p* values were added to the figure legends.

#### 3D Distances From Each Allele to the INM

The distance between each allele and the INM was measured in Imaris. By using the surface function and choosing the automated creation option, we created the INM surface. The distance between spots and the created INM surface were then computed using the “distance between spots and surfaces” function in Imaris and the resulting values returned by Imaris were pasted into Excel for the graphical display (**Data Sheet 7** and **Table 8**, in supplement).

#### Nuclear Volume Measurements

Nuclear volume measurements were performed in Imaris by using the “surface” function and choosing the automated creation option. Nuclear volumes could then be read in the statistics tap of the created surfaces under the “detailed” tap choosing “volumes.” In the list of all volumes displayed (in µm³), the highest value highlighted the nucleus in yellow; if other surfaces were also highlighted in yellow—which could sometimes occur later in the time course—the time point was excluded from the Excel file. Consequently, in supplementary **Data Sheet 7** there are missing data points of volume measurements toward the end of the experiment (**Table 9** in supplement).

## Results

### Changes in Fluorescence Intensity of Membrane-Associated and Soluble SEpHluorinD in Root Cells in Response to eATP

#### Tool Improvement

Constructs used in this study are shown in supplementary **Data Sheet 1**. Improvements made since a previous study (Matzke and Matzke, 2015) in the construct collection include using the *RPS5A* promoter (At3g11940) (Weijers et al., 2001) instead of the UBI-10 promoter (At4g05320) (Grefen et al., 2010) to drive expression of SEpHluorinD and other fluorescent proteins, and targeting SEpHluorinD to additional cellular compartments, including the cytoplasm, nucleoplasm and INM facing the nucleoplasm. Of these, the clearest localization of SEpHluorin was observed at the cytoplasmic face of the PM, the INM facing the perinuclear space, and the nucleoplasm. Hence, these locations were used in the present study. Correct membrane localization and responses of SEpHluorinD in the PM and INM to 2 mM eATP application in root cells has been documented previously (Matzke and Matzke, 2015) and has been confirmed and expanded in this study, which also tested 100-mM ATP (**Data Sheet 2** in supplement). The *RPS5A* promoter gives relatively reliable expression in the root tip region, particularly in the transition zone, which was the region examined in this work. The transition zone is important for perceiving environmental signals and hormone crosstalk (Kong et al., 2018) and was regarded by Charles Darwin as the “brain” of the root (Baluška et al., 2009).

#### Experimental Support for the Hypothesis Using New Constructs

Experiments using SEpHluorinD targeted to the PM, the INM facing the perinuclear space, and the nucleoplasm indicated that eATP induces immediate and contemporaneous decreases in pH (i.e. the fluorescence of SEpHluorinD diminishes) at all cellular locations tested (PM, cytoplasm, ONM, INM and nucleoplasm) (**Data Sheet 2** in supplement, and **Figure 2D**). Following the initial sharp decreases in pH, the further timing of the pH reductions was similar in all locations, with the lowest level occurring around 2 min after eATP application followed by a gradual reapproach to the baseline over the next 15 min. The maximum decrease in pH was estimated from the changes in SEpHluorinD fluorescence intensity to be approximately 0.9 pH unit (**Data Sheet 3** in supplement). Treatment with both 2 mM and 100 mM eATP elicited similar responses, indicating that ATP acts over a broad concentration range in this system. For unknown reasons, the baseline was exceeded during the recovery phase after application of 100 mM ATP in some cases (**Data Sheet 2** in supplement).

### Analysis of Chromatin Dynamics

#### Tool Improvement

To study 3D interphase chromosome arrangement in *Arabidopsis* roots, we previously used a collection of 16 distinct transgenic lines, each of which harbors a unique genomic site fluorescently tagged with either the Lac system or the Tet system (Matzke et al., 2005; **Data Sheet 4** in supplement). Although the *lac* or *tet* operator (*lacO or tetO*) repeats are stably integrated at these different genomic sites, the genes encoding the repressor protein-fluorescent protein (RP-FP) fusion proteins (*TetR-EYFP* and either *LacR-dsRed2* or *EGFP* under the control of the 35S promoter), which are on the same T-DNA construct as the operator repeats (**Data Sheet 4** in supplement), have become silenced over time. Therefore, to adapt these lines for studying chromatin dynamics in the experiments reported here, the desired RP-FP fusion proteins were supplied in trans from a second T-DNA that was introduced into the operator-repeat containing lines by super-transformation using a different selection marker (**Data Sheet 1**). In addition to gene silencing, a further problem with the original lines was the presence of EYFP (Tet system) and EGFP or dsRed2 (Lac system) in the RP-FP fusion proteins. These FPs contain intact dimerization domains, which can lead to unwanted protein-mediated chromosome pairing of repetitive operator arrays (Mirkin et al., 2013). Therefore, to avoid aberrant protein-mediated pairing of tagged genomic sites during studies of chromatin dynamics, monomeric mCitrine was used in the new Tet-R construct instead of EYFP, and monomeric mRuby was used in the new Lac-R constructs instead of EGFP and dsRed2 (Supplementary **Data Sheet 1**, combination **Figure 1**, in supplement).

To demonstrate the feasibility of using the improved fluorescence tagging system to simultaneously study the dynamics of unlinked and distinctly colored genomic sites, we used an Arabidopsis line harboring the homozygous loci 5:106 and 16:112, which contain *tetO* and *lacO* repeats integrated on the top and bottom arms of chromosome 5, respectively. The repeats bind Tet-repressor (TetR)-mCitrine and mRuby-Lac-repressor (LacR) fusion proteins that are encoded on a second T-DNA, resulting in strong, differently colored fluorescent signals at the two tagged chromatin sites (**Figures 1A, B**). Data from a pilot experiment of chromatin dynamics in the root tip region of untreated seedlings are shown in **Figure 1C**. The graph is based on the analyses of the cumulative 3D distance changes between the alleles in 10 nuclei during 10 min; the Δd^2^(μm^2^) values obtained for each nucleus were averaged and displayed in the graph. When calculated as an order 2 polynomial trendline, the results suggested that in 10 sampled nuclei in adjacent cells, locus 5:106 (green) was on average more dynamic than locus 16:112 (red). Standard deviations of the plotted average values of Δd^2^ were relatively high. However, an examination of individual nuclei revealed inter-nucleus variability. Nine (90%) of 10 nuclei displayed a statistically significant difference (*p* < 0.05) in mobility between locus 16:112 and locus 5:106, and of these, six (60%) followed the average trend of higher mobility at locus 5:106 (**Figure 1C**).

These findings suggest that distinct loci in the same nucleus may frequently exhibit variations in the degree of chromatin mobility (“jiggling”; **Video 3** in supplement). By contrast, fixed, nonviable seedlings show little movement (**Figures 1D, E**).

#### Experimental Support for the Hypothesis

##### 1. Chromatin Dynamics Following Addition of eATP

We also investigated chromatin dynamics before and after addition of eATP during data acquisition. For this analysis, we used a line harboring locus 16:101, which contains homozygous *lacO* repeats on the bottom arm of chromosome 1, and a second T-DNA that encodes INM-localized pH-sensitive SEpHluorinD and an mRuby-LacR fusion protein, which is insensitive to pH (**Data Sheet 1**; combination **Figure 2**, in supplement). By observing the fluorescence intensity changes of INM-localized SEpHluorinD, we can verify that eATP treatment has an effect on pH at the nuclear rim and at the same time, monitor dynamics of the *lacO* sites colored with mRuby-LacR protein in multiple nuclei (**Figures 2A and B**). An order 2 polynomial trendline of average Δd2 values for 10 nuclei at each time point suggested generally reduced chromatin dynamics (pink line) of locus 16:101 after eATP treatment compared to before eATP exposure (black line) during the 30 min of data acquisition [i.e. Δd^2^(μm^2^) plateaus at approximately 0.10 before ATP treatment and approximately 0.07 after ATP addition] (**Figure 2C**). Examination of individual nuclei again revealed inter-nucleus variability. Seven of ten (70%) of the sampled nuclei displayed statistically significant (*p* < 0.05) changes in chromatin mobility, and of these, six exhibited reduced mobility of locus 16:101 after eATP treatment, and are thus consistent with the trend seen in the averaged results (**Figure 2C**).

A concomitant drop in fluorescence intensity followed by partial leveling off of INM-localized SEpHluorinD upon application of eATP demonstrated that this stimulus was effective in inducing a pH change at the nuclear periphery (**Figure 2D**). As expected, the pH-insensitive mRuby-LacR chromatin tag did not exhibit a drop in fluorescence intensity following eATP treatment, only a continuous and irreversible decline (**Figure 2E**) similar to that seen with the buffer-only control (**Figure 3**). (Patterns of reductions in fluorescence are described in the *Materials and Methods* section.) These results supported the idea that the drop in fluorescence intensity observed with INM-localized SEpHluorinD depends on the pH sensitivity of this protein.

A significantly different change in mobility following eATP addition was also observed in a certain percentage of nuclei at other loci tested 'locus 16:112 (**Figure 3C**; **Table 6**); and locus 5:106 (**Data Sheet 6C** in supplement and **Table 7**)'. Collectively, the findings on three loci support a correlation in at least some nuclei between concurrent changes in chromatin dynamics and nuclear pH.

##### 2. Can eATP-Induced pH Changes Be Sensed by Chromatin Proteins?

The experiments described above demonstrate coordinated and transient changes in pH in close proximity to the PM and INM and in the nucleoplasm subsequent to application of eATP (**Data Sheet 2** in supplement). Moreover, the pH changes elicited by eATP could be temporally correlated with changes in chromatin motion in the nucleus (**Figure 2**). An interesting follow-up question is whether these pH changes are felt directly by chromatin-associated proteins, which would be important for pH-dependent regulation of chromatin structure and gene expression. Alternatively, chromatin constituents may be insulated from surrounding changes in pH and fail to respond to eATP.

To investigate this question, we assembled a construct encoding a SEpHluorinD-LacR fusion protein together with PM and INM-targeted mApple, which served in this experiment as red visual markers for these two membrane systems (**Figures 3A, B**; **Data Sheet 1**, combination **Figure 3**, in supplement). Through the LacR domain, the RF-FP fusion protein can bind to the *lacO* repeats integrated into the genome and through the SEpHluorinD moiety, the RP-FP fusion protein can respond to pH changes as revealed by alterations in fluorescence intensity. If eATP-induced changes in nuclear pH affect chromatin directly, then eATP should elicit fluorescence changes in chromatin-bound SEpHluorinD. The construct was used to super-transform a line harboring locus 16:112, which contains homozygous *lacO* repeat arrays on the bottom arm of chromosome 5 (**Figure 3A**, right). In a pilot experiment using this line, eATP treatment elicited not only a statistically significant (*p* < 0.05) change in chromatin dynamics in 30% of sampled nuclei (**Figure 3C**), but also a relatively rapid reduction (occurring over a period of approximately 3 min in individual nuclei) followed by a partial leveling off in the fluorescence intensity of SEpHluorin-LacR at both 16:112 alleles. These findings indicate a drop in pH directly at the corresponding genomic sites. This pattern was particularly visible in nuclei 1, 2, 3, 6, 7, 8, 9 and 10 (blue boxed, **Data Sheet 8**, parts “**Figure 3D** and **Figure 3E**” in supplement). Addition of buffer did not induce a comparable response (**Figure 2E**, **Figure 4A and B**, and **Figure 5C**, right), consistent with the observed decrease in fluorescence being dependent on eATP. A similar lack of response was observed with pH-insensitive RP-FP fusion proteins mRuby-LacR and mCitrine-LacR following eATP treatment (**Figures 2E**, **4**, and **5C**, **right**; see individual nuclei in corresponding parts of **Data Sheet 8** in supplement). These findings demonstrate that the behavior of SEpHLuorinD-LacR in this experiment is dependent on its pH sensitivity. From the magnitude of the decrease in SEpHluorinD fluorescence, the maximum reduction in pH at locus 16:112 was estimated to be approximately 0,4 pH units (**Data Sheet 3** in supplement).

To substantiate the observed effects of eATP on the pH at a specific genomic site, a modified experiment was performed using pH-sensitive and pH-insensitive chromatin tags bound at the same locus. For this, a construct was assembled that encoded two RP-FP fusion proteins: SEpHluorinD-LacR and mRuby-LacR (**Data Sheet 1**, combination **Figure 5**, in supplement), which represent, respectively, pH-sensitive and pH-insensitive proteins that bind to *lacO* repeats (**Figure 5A**). After introducing this construct into a line harboring homozygous locus 16:112, two yellow fluorescent dots arising from co-localization of the red and green RP-FP fusion proteins at both 16:112 alleles were observed in root tip nuclei (**Figure 5B**). Reproducing the results in **Figure 3D**, treatment with eATP in a pilot experiment resulted in a relatively rapid decrease (approximately 7 min) followed by a partial leveling off in the fluorescence of the SEpHluorinD-LacR in all nuclei observed (**Figure 5C**, **left**). The decrease in fluorescence intensity following eATP addition was observed at each allele of locus 16:112, consistent with a coordinate drop in pH directly at both alleles of this locus. At the same time, no comparable drop in fluorescence intensity of the mRuby-LacR tag at either of the two alleles of locus 16:112 was observed, only a continuous decline in fluorescence consistent with bleaching (**Figure 5C**, **right**). The results suggest that the relatively rapid response of SEpHluorinD-LacR to eATP is not a general reaction of RP-FP fusion proteins but depends on the pH sensitivity of SEpHluorinD. The collective findings support the hypothesis that an extracellular stimulus that triggers changes in nuclear pH can also incite concurrent changes in the surrounding pH of chromatin-associated proteins.

## Discussion

We have developed and used improved tools to obtain support for the existence of a rapid, ion-based signaling pathway that initiates at the cell surface and reaches chromatin in the nucleus to impact interphase chromosome dynamics and chromatin-bound proteins. Although experimentally induced by eATP in our system, such an ion-based pathway could possibly operate in natural settings to allow external signaling molecules and environmental stimuli to quickly adjust gene expression by changing the structures and/or activities of chromatin proteins. Moreover, interconnected electrical/ionic-based signaling pathways could exert an overall patterning effect on cellular constituents at the subcellular, cellular and supra-cellular levels by altering the spatial and temporal distribution of ions sequestered at the charged surfaces of membranes and in membrane-bound compartments.

It is not clear how pH changes at the PM, which are induced by an extracellular signal that presumably does not enter the cell, are transmitted to the nuclear membranes and nucleoplasm to influence the properties of chromatin. When considering the series of events involved in the observed pH changes, it is important to separate the pH changes themselves, which generally take approximately 2 to 7 min to reach a maximum in our experiments, from the immediate and simultaneous initiation of these changes at the PM and INM following eATP exposure. The genomic sites tagged with a pH-sensitive DNA-binding protein also exhibited decreased SEpHluorinD fluorescence following similar kinetics. Since the pH changes at all three locations are highly synchronized, one speculation is that an electrical signal is rapidly conveyed from the PM to the nucleus through internal membranes. This electrical signal could trigger the opening or closing of H^+^ channels in the INM and subsequent release of protons from the perinuclear space and/or INM surfaces, resulting in pH-dependent changes in the properties and behavior of chromatin. Although the bulk pH of the cytoplasm, nucleoplasm and ER in plant cells has been estimated using a pH-sensitive fluorescent protein to range from 7.0 to 7.4 (Shen et al., 2013), localized membrane surface pH and other micro-domains of pH within a cell can differ from bulk pH in ways that are physiologically relevant (Maouyo et al., 2000; Felle, 2001). If a particular locus is situated peripherally in the nucleus, it could fall under the influence of a variable INM surface pH. Alternatively, protons, which are highly mobile in plant cells (Felle, 2001), could diffuse rapidly away from the INM after release from the perinuclear space and enter the nucleoplasm to affect not only peripheral but also more centrally located chromatin sites. The pH of the perinuclear space *per se* has not been determined, but existence of H^+^ pumps in NMs of at least some cell types (Santos et al., 2016) suggests the possibility of regulated transport of protons from the perinuclear compartment into and out of the nucleoplasm.

### Influence of pH Changes on Chromatin

Changes in pH can conceivably bring about alterations in chromatin mobility and function in different ways. For example, chromatin “jiggling,” which appears to be evolutionarily conserved among eukaryotes, is largely attributed to the activity of ATP-dependent chromatin remodeling complexes that continually open and close condensed chromatin fibers to regulate access of the transcriptional machinery to genomic DNA (Soutoglou and Misteli, 2007; McNally, 2009). The activities of chromatin remodelers and other transcriptional proteins are likely to be sensitive to differences in the local pH. Consistent with this supposition, our results demonstrate that a fluorescent DNA-binding protein can directly perceive changes in the surrounding pH and undergo alterations that affect fluorescence intensity (Campbell and Choy, 2001). Chromatin mobility and function could also be influenced indirectly through pH-induced alterations of nucleoskeletal elements. The integrity and stability of the actin-containing nuclear matrix, which helps to organize chromatin and facilitate chromatin remodeling and transcription, are known to be sensitive to changes in pH values (Libertini and Small, 1984; Wang et al., 1989).

### Inter-Nucleus Variability

The considerable variability in our data appears to reflect natural heterogeneity that is increasingly recognized as an inherent feature among individual cells of a given population (Argueso et al., 2019; Nicholson, 2019). Although average values can suggest overall trends in the data, they can mask real and potentially important differences at the single cell/nucleus level (Argueso et al., 2019; Nicholson, 2019). Indeed, examination of our data on the level of single nuclei revealed that all nuclei within a given group of cells did not respond identically to eATP treatment. In the experiments examining chromatin dynamics, examples of reduced, increased and unchanged motion can be observed in nuclei of neighboring cells. Similarly, pH-sensitive chromatin bound proteins did not respond identically to eATP in all sampled nuclei of a given root. The observed heterogeneity may depend on a number of factors, including fluctuations in the physiological status of individual nuclei during the period of data acquisition, and varying locations of different chromatin sites in 3D nuclear space. An additional source of variation may arise from the exact position in the root and identity of the cells sampled. Although we aimed to study cells in microscopically visible layers of the transition zone, this region does not have clearly defined borders to the adjoining elongation zone or meristematic region. Heterogeneity in individual cells of a population could conceivably enable plants to adapt more effectively to a constantly changing environment by providing alternative physiological states that offer a range of advantageous properties.

### Reproducibility of Results

Although the experiments have been carried out using different transgenic plant lines expressing various fluorescent proteins, the findings display a high degree of internal consistency. For example, in experiments examining fluorescence intensity changes of pH-insensitive fluorescent proteins, two different pH-insensitive fluorescent chromatin-binding proteins (mRuby-LacR and mCitrine-LacR) bound to two different loci (locus 16:101 and locus 16:112) showed similar responses following eATP addition in three different experiments (**Figures 2E**, **4C**, and **5C** **right**). In addition, two different pH-sensitive fluorescent fusion proteins (SEpHluorinD fused to either LacR or SUN2) exhibited similar behavior following eATP addition in three different experiments (**Figures 2D**, **3D** **left**, and **5C** **left**). Finally, in experiments to assess changes in chromatin dynamics, three different fluorescent chromatin-binding proteins (pH-sensitive SEpHluorinD-LacR and TetR-SEpHluorinD) and a pH-insensitive fluorescent chromatin-binding protein (mRuby-LacR) that bound, respectively, to three different loci (locus 5:106, locus 16:101 and locus 16:112) displayed altered chromatin dynamics following eATP treatment in three different experiments (**Figures 2C** and **3C**, and **Data Sheet 6C**, in supplement). These consistent trends among experiments reinforce the reproducibility of our results.

### Limitations of Experimental System

The set of tools described here can be used in closely monitored experiments to obtain dependable results on 3D interphase chromosome mobility and pH at specific genomic loci and other cellular locations. That said, further improvements and developments of tools and imaging technology will be required in the future to overcome several limitations of the current experimental system (Lobet, 2017). For example, despite the use of the more reliable *RPS5A* promoter in the improved constructs, the expression level of the RP-FP fusion proteins in root cells is still not completely stable and fluorescent signals from chromatin-tagged sites are often weak and non-uniform within a single seedling and among sibling seedlings. Therefore, before performing confocal microscopy, it remains necessary to screen seedlings under a stereo-fluorescence microscope to identify those with the strongest signals in the root tip. Even in plants originally exhibiting strong fluorescent signals from a tagged locus, this phenotype is often not inherited in progeny, probably due to frequent silencing of genes encoding RP-FP fusion proteins (Matzke et al., 2010). Hence, the most intense signals are usually observed in primary transformants. Further improvements in constructs (for example, finding and using promoters less susceptible to silencing) will facilitate more uniform expression in primary transformants and consistent heritability of strong expression in subsequent generations. These advances will allow the establishment of stable transgenic lines that can be used repeatedly for experiments.

Treatment of seedlings mounted on slides with eATP is tricky and it is difficult to avoid introducing a degree of uncertainty into the experiments. Although the method described here (adding the ATP solution to the seedling root through a perforation in the cover slip) can be done relatively quickly, it is not possible with this technique to determine the actual concentration of available ATP present at the root tip. However, as our results indicate, eATP works over a wide concentration range in this system (**Data Sheet 2** in supplement), so some variation in eATP concentration around the root tip can be tolerated. Addition of eATP (or buffer) using this method also leads to transient “dislocation turbulence” (up and/or down displacement of the root) (Supplementary **Videos 1** and **2**). The use of a microfluidic chip (Grossmann et al., 2018) might alleviate these problems in future studies.

Our system is versatile and potentially allows many other treatments to be examined such as electrical pulses, different hormones, temperature shifts, mutations in signal transduction components, and various ionophores and ion channel blockers. To study the proposed pathway in other cell types, different tissue-specific promoters can be used to drive expression of RP-FP fusion proteins and SEpHluorinD derivatives. Additional targeting signals can be used to direct SEpHluorinD to other cellular locations (e.g., the ER, organelles, outer surface of PM). The eventual development of brighter and more photo-stable FPs that have unique excitation and emission spectra in combination with more sensitive microscopes will be useful for simultaneous visualization and data acquisition from multiple fluorescent sensors within the cell.

### Future Perspective

Although these experiments can only be considered initial tests of the hypothesis, the overall findings generally support the existence of a highly efficient and interconnected electrical/ion-based signaling pathway extending from the PM to the nucleus, possibly through intervening membrane systems, which can influence chromatin behavior. The experiments reported here relied on fluorescent proteins that are sensitive to pH and not directly to membrane voltage. Although pH changes can be a downstream consequence of voltage changes, a more definitive test of the proposed pathway awaits the development of GEVIs that reliably respond to shifts in voltage at multiple membrane systems in plant cells. Developing such tools is an important goal for the future (Matzke and Matzke, 2015; Basu and Haswell, 2017). In support of the existence and evolutionarily conservation of this hypothetical pathway, a recent study using an ER-localized ArcLight derivative in human cells indicated that electrical signals at the PM can affect the voltage of internal membranes (Sepheri-Rad et al., 2018).

## Data Availability Statement

All datasets generated for this study are included in the manuscript/**Supplementary Files**.

## Author Contributions

AM and MM were involved in all aspects of this work. W-DL contributed to statistical evaluation of the data.

## Funding

Funding for this project was provided by Academia Sinica (AS) and the Institute of Plant and Microbial Biology at AS.

## Conflict of Interest

The authors declare that the research was conducted in the absence of any commercial or financial relationships that could be construed as a potential conflict of interest.

## Supplementary Material

Supplementary Video 1 Dislocation turbulence_Figure3C_front view.avi: locus 16:112 (green), 30 minutes (in Video reduced to 6 secs: 5 acquisition points /sec, +ATP at time point 13 during red channel acquisition), PM and INM colored in red (CBL1-mApple and SUN2-mApple respectively)

Supplementary Video 2 Dislocation turbulence_ side view of Video1

Supplementary Video 3 Chromatin ‚jiggling‘ Figure1B_right.avi : locus 5:106 (green), locus 16:112 (red), 10 minutes (in Video reduced to 2 secs: 5 acquisition points/sec)
