# Supplementary material for: Evidence That Ion-Based Signaling Initiating at the Cell Surface Can Potentially Influence Chromatin Dynamics and Chromatin-Bound Proteins in the Nucleus
Source: Front Plant Sci. 2019 Oct 17;10:1267. doi: 10.3389/fpls.2019.01267 (PMC6811650; doi:10.3389/fpls.2019.01267)
Supplement: Supplementary file 4 [file DataSheet_1.pdf]

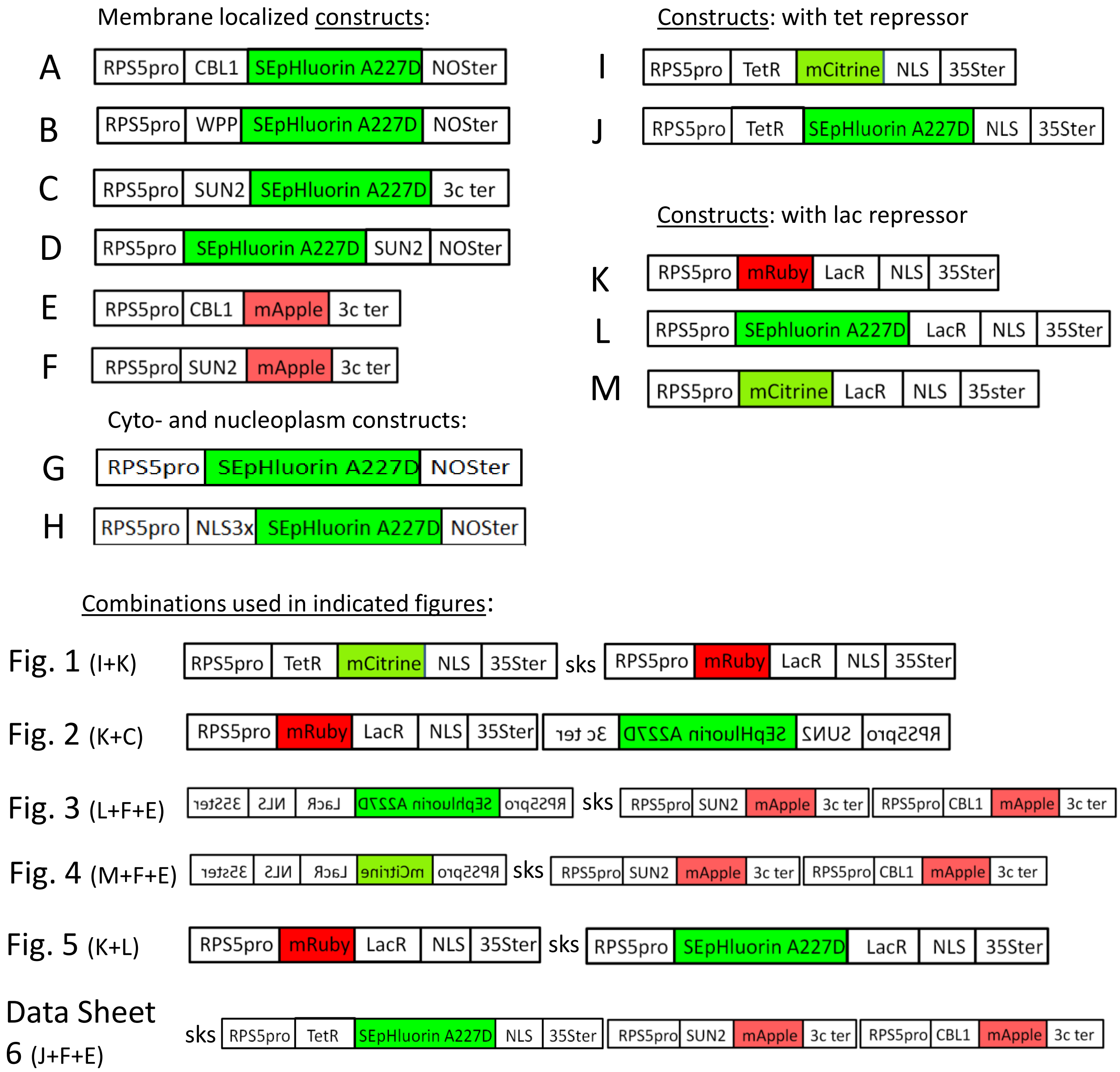

pH sensitivity of fluorescent proteins

mCitrine – pH-insensitive (Griesbeck et al., 2001)
SEpHluorinA227D – pH-sensitive (Matzke and Matzke, 2015)
mRuby – pH-insensitive (Kredel et al., 2009)
mApple – pH-sensitive, but fast bleaching (Shaner et al., 2008)

Data Sheet 1 Matzke et al

Data Sheet 1: Constructs and pH sensitivity of fluorescent proteins used in this study

Constructs were produced avoiding *Sal*I, *Xho*I and *Sac*II sites – if necessary having DNA synthesis carried out by Genscript (www.genscript.com) using standard molecular biology techniques. Building block constructs A-L were assembled on modified pBC plasmids (Stratagene, Cat. Nr. 21215) between *Sal*I and *Xho*I sites. *Sal*I/*Xho*I digestion releases the construct from the pBC plasmids. The released construct was ligated into the *Sal*I site of binary vector pPZP221 with a 35Spro-driven gentamicin selection marker (Hajdukiewicz et al., 1994). The above binary vector constructs can be cut again with *Sal*I and for construct combinations, more *Sal*I/*Xho*I assembled constructs can be added. Tagging the *Sal*I/*Xho*I assembled construct with *Sal*I *Sac*II KAN *Sac*II *Xho*I (abbreviated as 'sks' in the combination constructs) allows direct selection for the combination constructs used for **Figs. 1-5**. The KAN selection marker can be deleted with *Sac*II (combination construct **Fig. 2**), and a third *Sal*I/*Xho*I assembled construct can be added (combination constructs **Figs 3** and **4**).

The respective binary vectors containing the desired transgene construct were introduced via Agrobacterium-mediated transformation into *Arabidopsis thaliana* using the floral dip method (Clough and Bent 1998). Seeds harvested from the dipped plants were surface sterilized and dispersed on solid Murashige and Skoog medium containing cefotaxime (200µg/µl) to eliminate agrobacteria and gentamicin (100µg/µl) for selection of transgenic plants (primary transformants).

Abbreviations: CBL1, a plasma membrane targeting motif (Batistic et al., 1998); SUN2, targeting motif for inner nuclear membrane (INM) Graumann et al., 2010, 2014); WPP, an outer nuclear membrane (ONM) targeting sequence (Deal and Henikoff, 2010); NOSter, nopaline synthase transcriptional terminator; 3Cter, transcriptional terminator from the *rbcS3C* gene (Benfey et al., 1989); 35Ster, transcriptional from cauliflower mosaic virus; *RPS5A* pro, promoter from the *RIBOSOMAL PROTEIN S5* (At3G11940) (Weijers et al., 2001); TetR, tetracycline repressor protein; LacR, Lac repressor protein; NLS, nuclear localization signal from SV40.

References

Batistic, O., Sorek, N., Schültke, S., Yalovsky, S., and Kudla, J. (2008). Dual fatty acyl modification determines the localization and plasma membrane targeting of CBL/CIPK Ca^{+2} signaling complexes in *Arabidopsis*. Plant Cell 20, 1346-1362.

Benfey, P.N., Ren, L., and Chua, N.H. (1989). The CaMV 35S enhancer contains at least two domains which can confer different development and tissue-specific expressioin patterns. EMBO J. 8, 2195-202.

Clough, S.J., and Bent, A.F. (1998). Floral dip: a simplified method for Agrobacterium-mediated transformation of *Arabidopsis thaliana*. Plant J. 16, 735–743.

Deal, R.B., and Henikoff, S. (2010). A simple methods for gene expression and chromatin profiling of individual cell types within a tissue. Dev. Cell 18,1030-1040.

Graumann, K., Vanrobays, E., Tutois, S., Probst, A.V., Evans, D.E., and Tatout, C. (2014). Characterization of two distinct subfamilies of SUN-domain proteins in Arabidopsis and their interactions with the novel KASH-domain protein AtTIK J. Exp. Bot. 65, 6499-6512.

Graumann, K., Runions, J., and Evans, D.E. (2010). Characterization of SUN-domain proteins at the higher plant nuclear envelope. Plant J. 61, 134-144.

Griesbeck, O., Baird, G.S., Campbell, E., Zacharias, D.A., and Tsien, R.Y. (2001). Reducing the environmental sensitivity of yellow fluorescent protein. J. Biol. Chem. 276, 29188–29194.

Hajdukiewicz, P., Svab, Z., and Maliga, P. (1994). The small, versatile pPZP family of Agrobacterium binary vectors for plant transformation. Plant Mol. Biol. 25, 989–994.

Kredel, S., Oswald, F., Nienhaus, K., Deuschle, K., Röcker, C., Wolff, M., Heilker, R., Nienhaus, G.U., and Wiedenman, J. (2009). mRuby, a bright monomeric red fluorescent protein for labeling of subcellular structures PLoS ONE 4, 1-7.

Matzke, A.J.M., and Matzke, M. (2015). Expression and testing of ArcLight, a genetically-encoded voltage indicator used in neuroscience research. BMC Plant Biol. 15: 245.

Shaner, N.C., Lin, M.Z., McKeow,n M.R., Steinbach, P.A., Hazelwood, K.L., Davidson, M.W., and Tsien, R.Y. (2008). Improving the photostability of bright monomeric orange and red fluorescent proteins. Nat. Methods 5, 545-551.

Weijers, D., Franke-van Dijk, M., Vencken, R.J., Quint, A., Hooykaas, P., and Offringa, R. (2001). An Arabidopsis Minute-like phenotype caused by a semi-dominant mutation in a RIBOSOMAL PROTEIN S5gene. Development 128, 4289-4299.
