# Supplementary material for: Evidence That Ion-Based Signaling Initiating at the Cell Surface Can Potentially Influence Chromatin Dynamics and Chromatin-Bound Proteins in the Nucleus
Source: Front Plant Sci. 2019 Oct 17;10:1267. doi: 10.3389/fpls.2019.01267 (PMC6811650; doi:10.3389/fpls.2019.01267)
Supplement: Supplementary file 5 [file DataSheet_2.pdf]

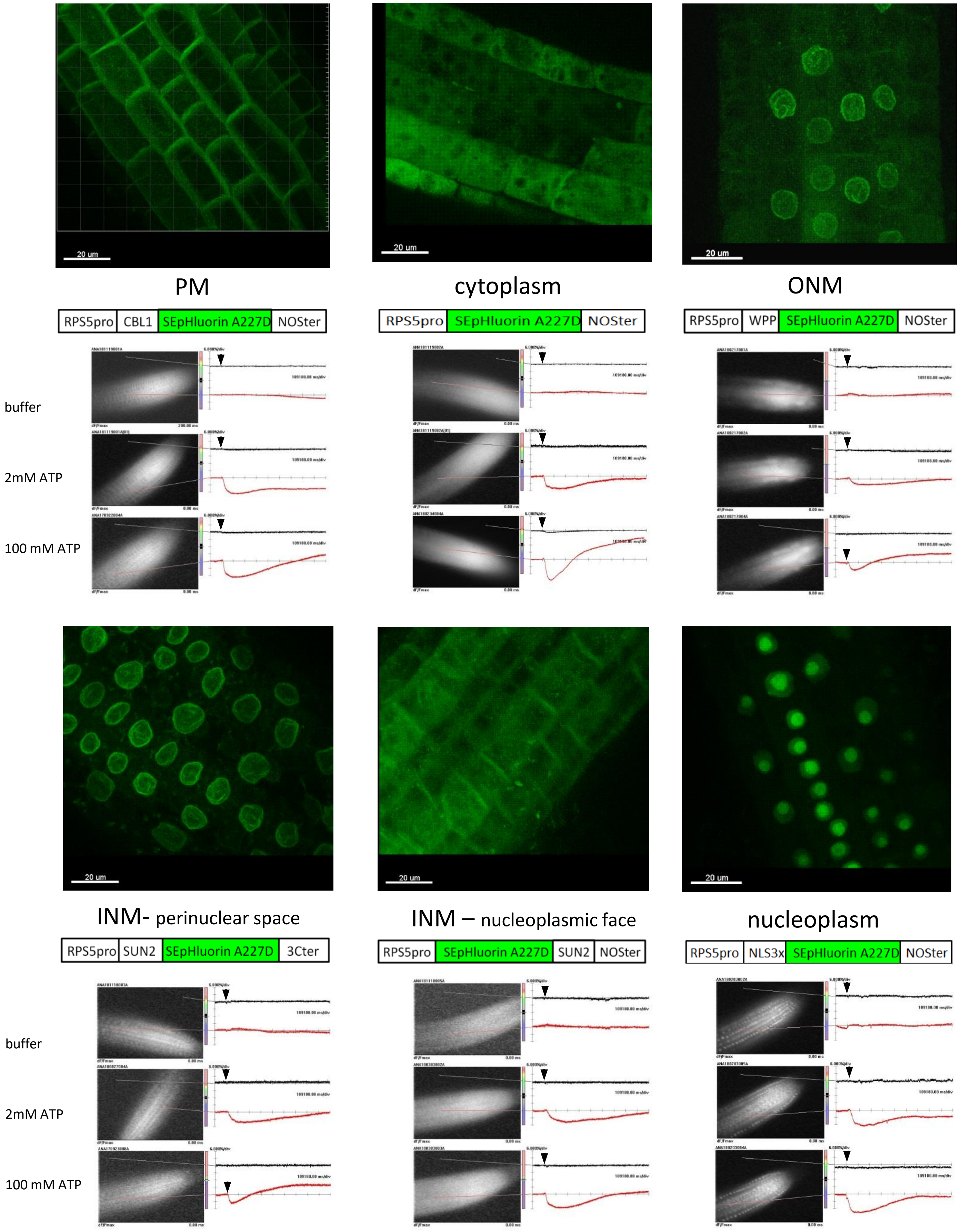

Dats Sheet 2 Matzke et al

PM

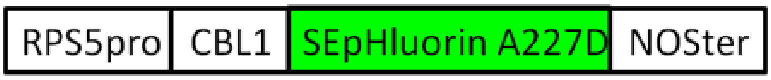
RPS5pro
CBL1
SEpHluorin A227D
NOSter

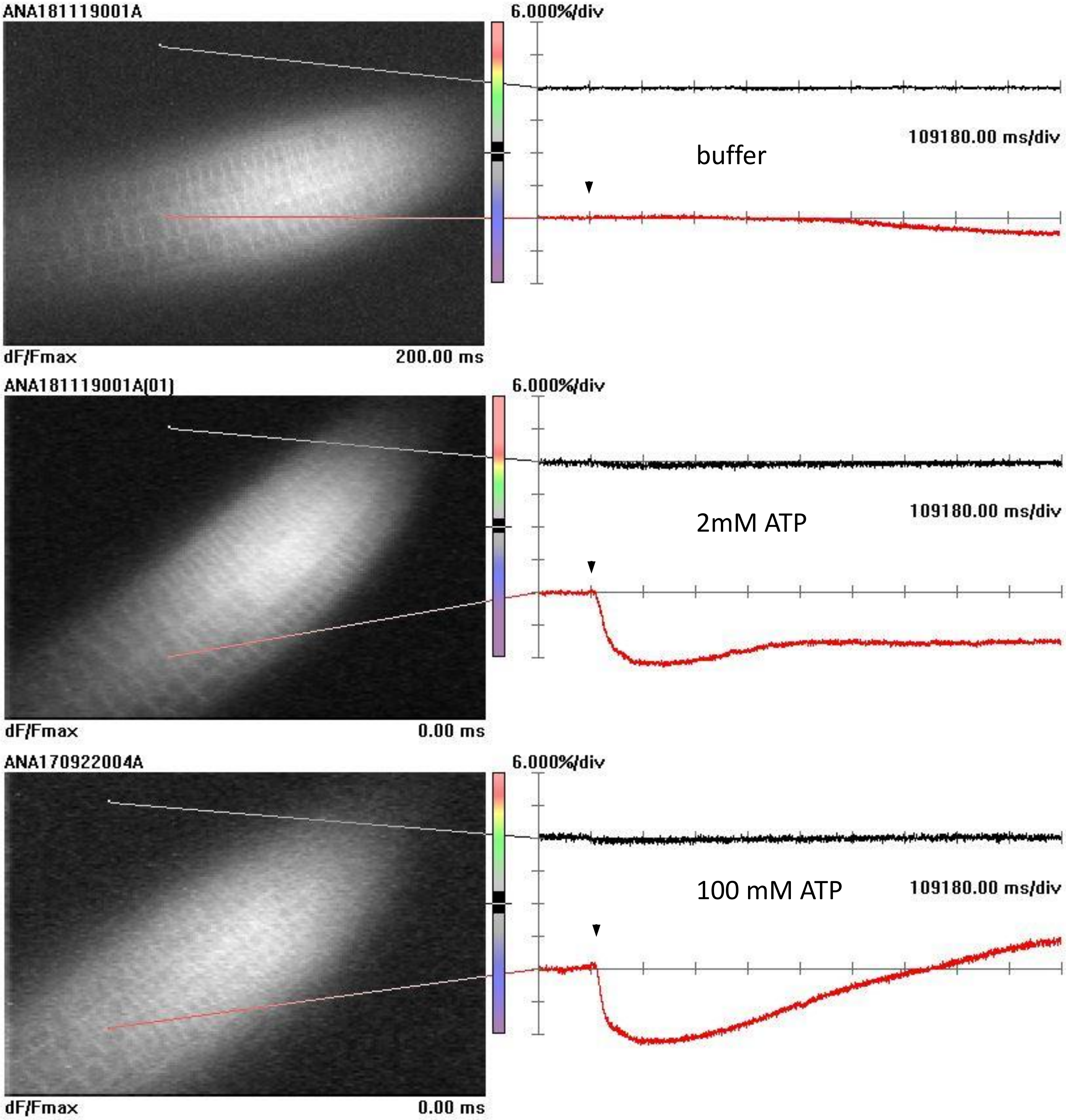
ANA181119001A
6.000%/div
109180.00 ms/div
buffer
dF/Fmax
200.00 ms
ANA181119001A(01)
6.000%/div
109180.00 ms/div
2mM ATP
dF/Fmax
0.00 ms
ANA170922004A
6.000%/div
100 mM ATP
109180.00 ms/div
dF/Fmax
0.00 ms

cytoplasm

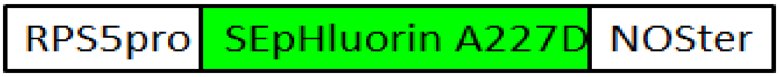
RPS5pro
SEpHluorin A227D
NOSter

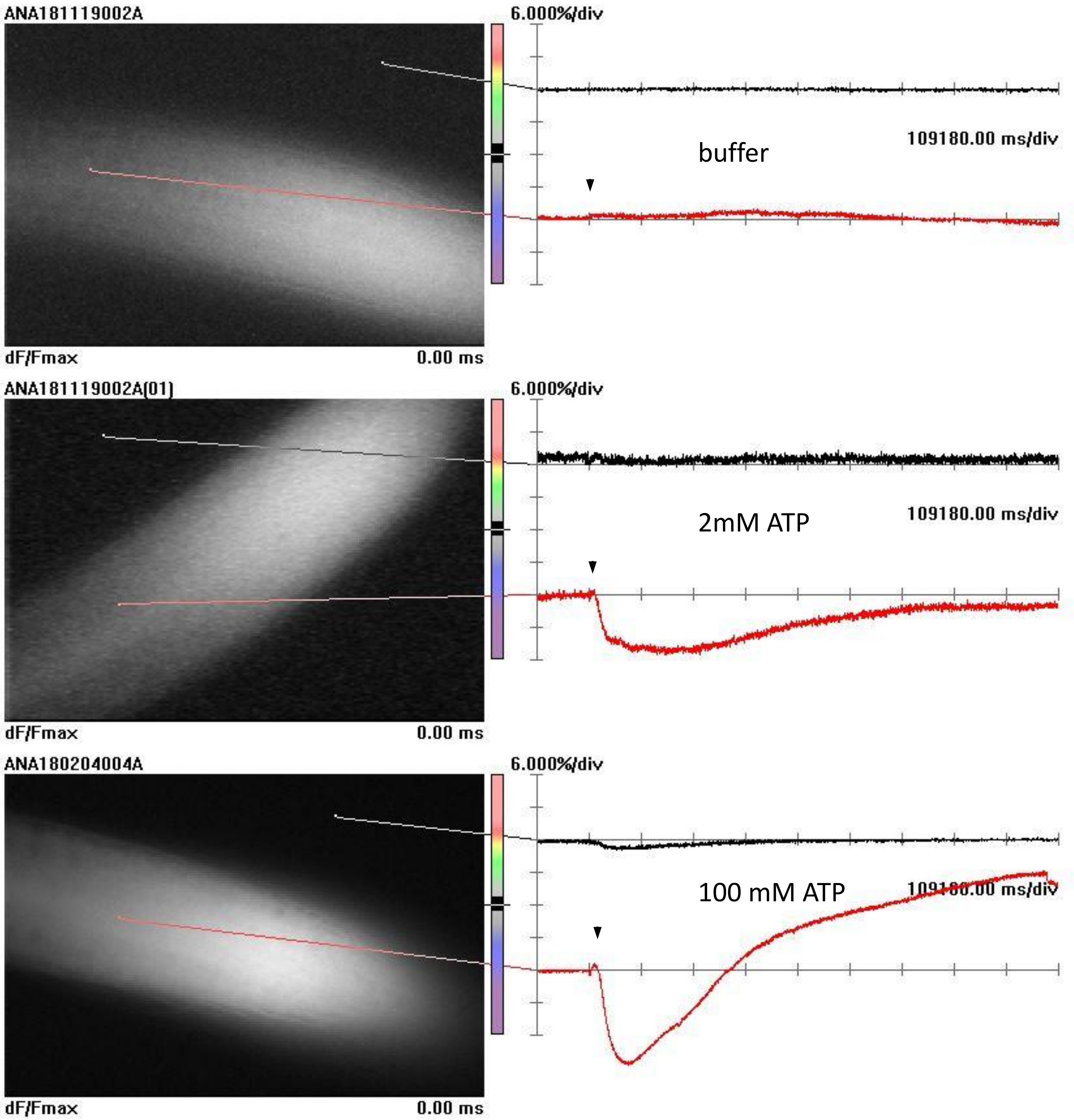
ANA181119002A
6.000%/div
109180.00 ms/div
buffer
dF/Fmax
0.00 ms
ANA181119002A(01)
6.000%/div
2mM ATP
109180.00 ms/div
dF/Fmax
0.00 ms
ANA180204004A
6.000%/div
100 mM ATP
dF/Fmax
0.00 ms

ONM

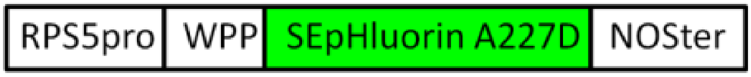
RPS5pro
WPP
SEpHluorin A227D
NOSter

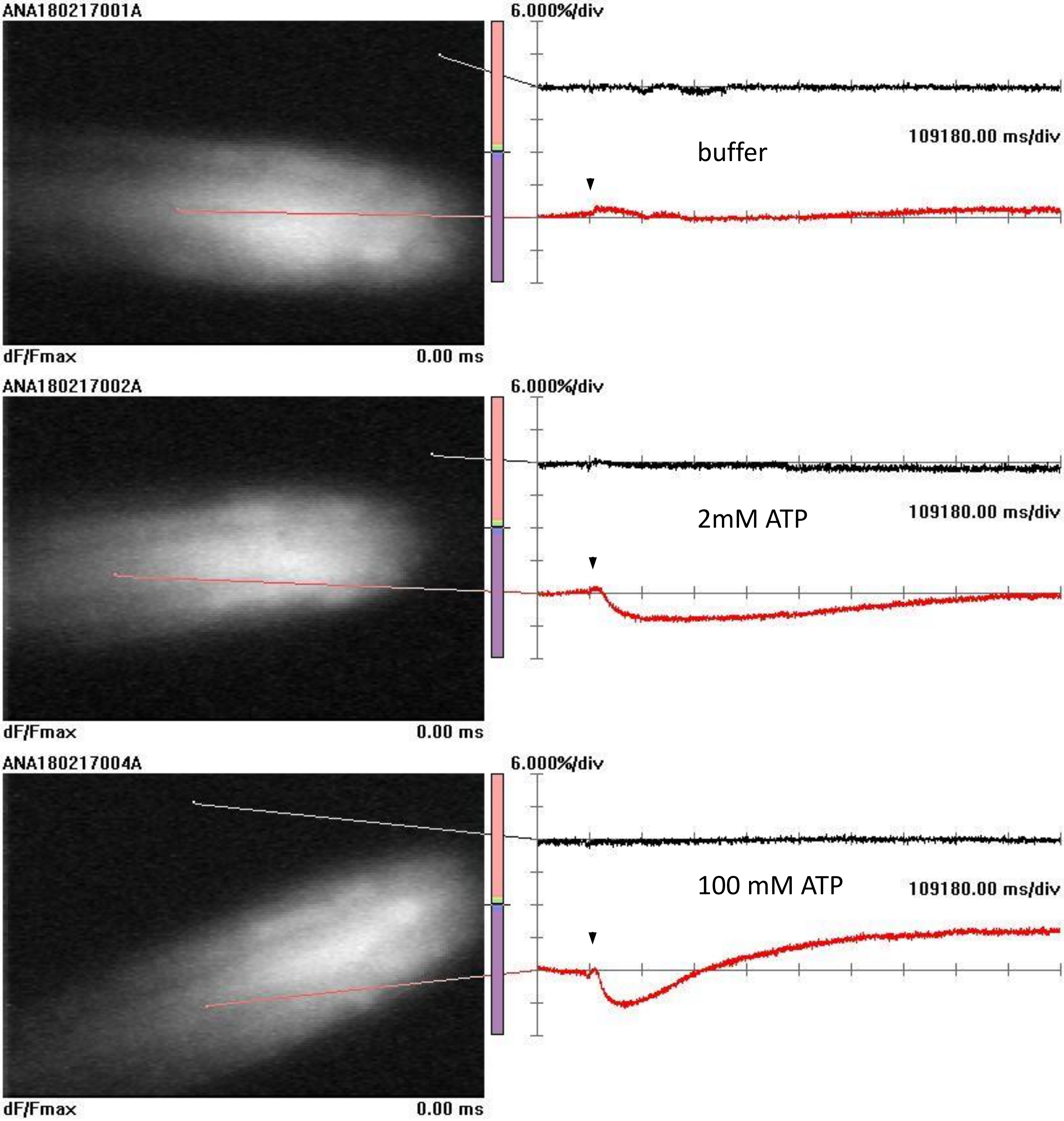
ANA180217001A
6.000%/div
109180.00 ms/div
buffer
dF/Fmax
0.00 ms
ANA180217002A
6.000%/div
2mM ATP
109180.00 ms/div
dF/Fmax
0.00 ms
ANA180217004A
6.000%/div
100 mM ATP
109180.00 ms/div
dF/Fmax
0.00 ms

INM perinuclear space

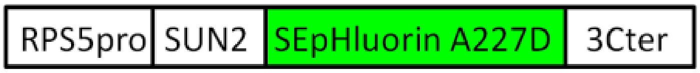

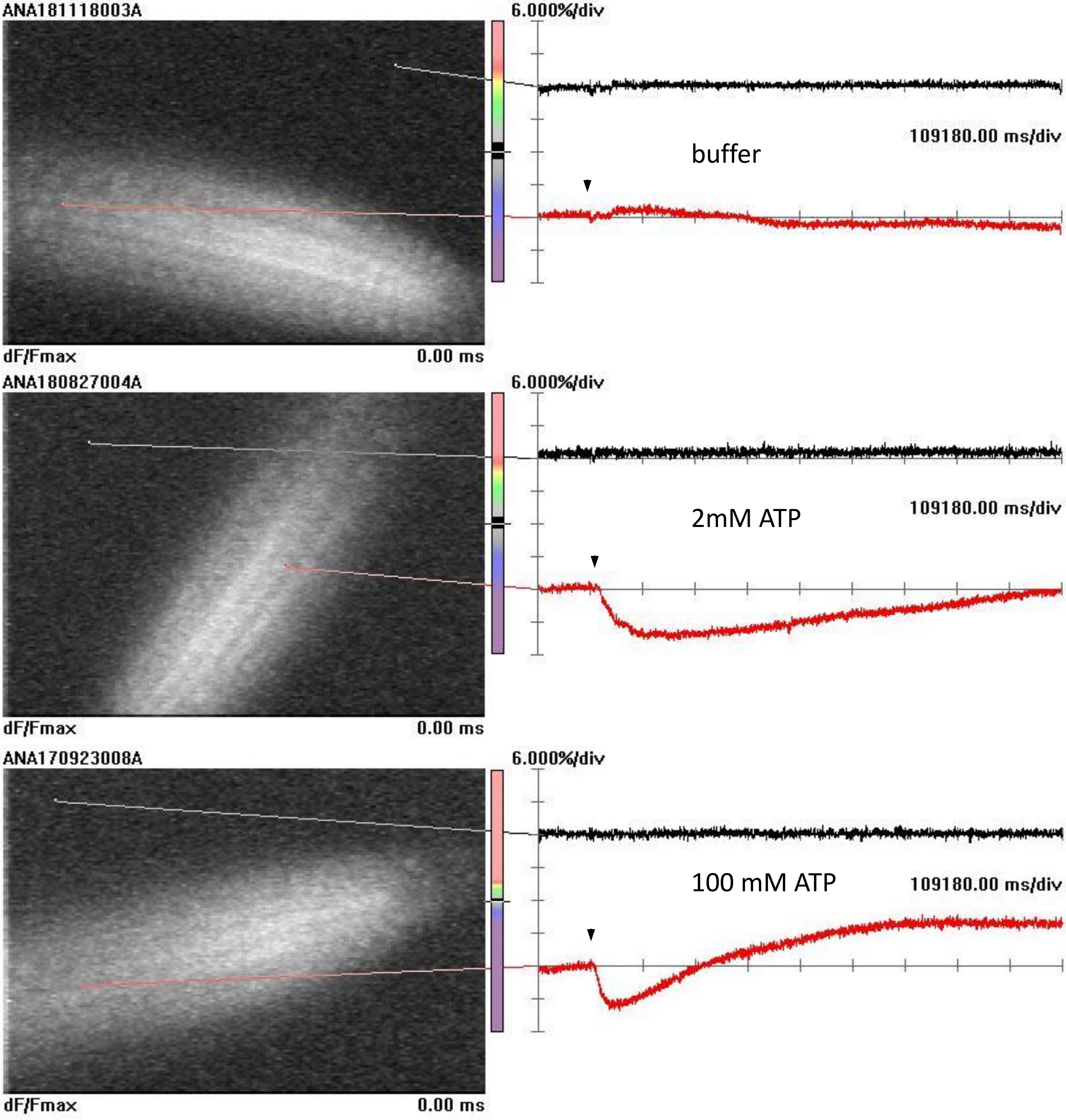

INM nucleoplasm

RPS5pro | SEpHluorin A227D | SUN2 | NOSter

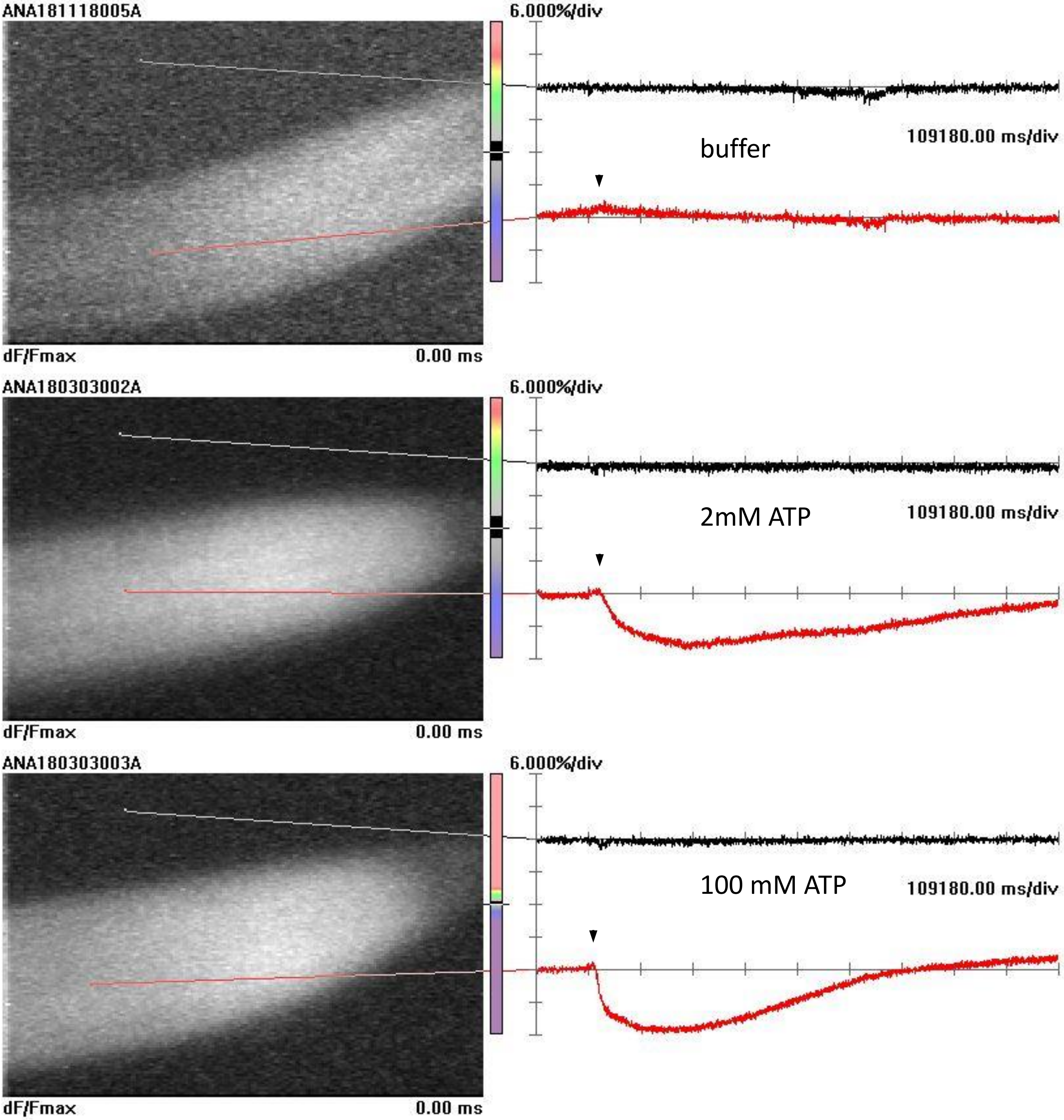

nucleoplasm

RPS5pro	NLS3x	SEpHluorin A227D	NOSter

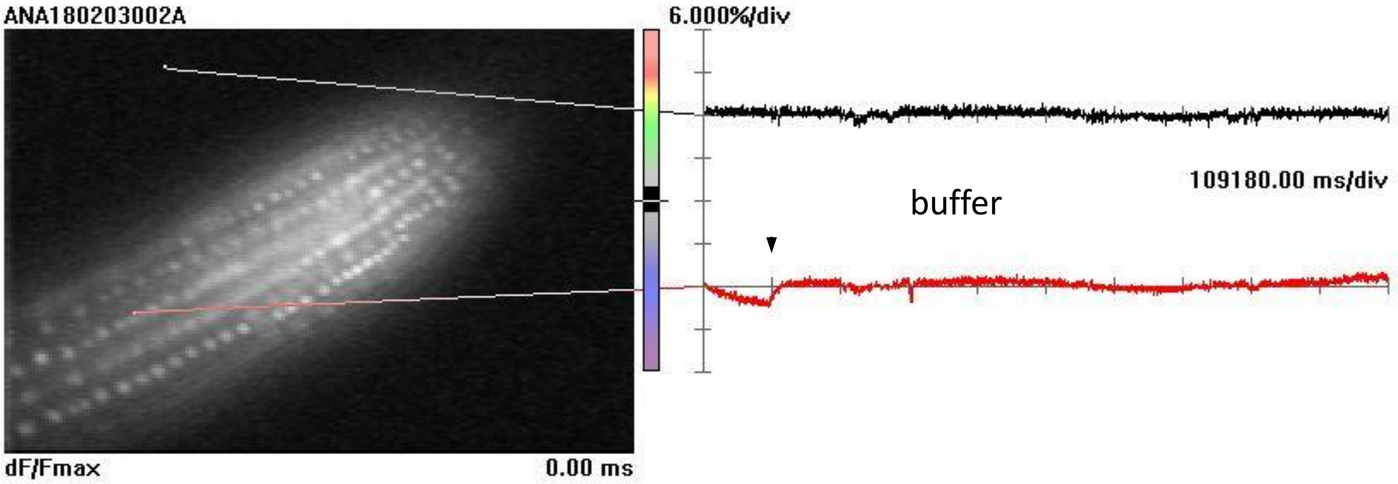

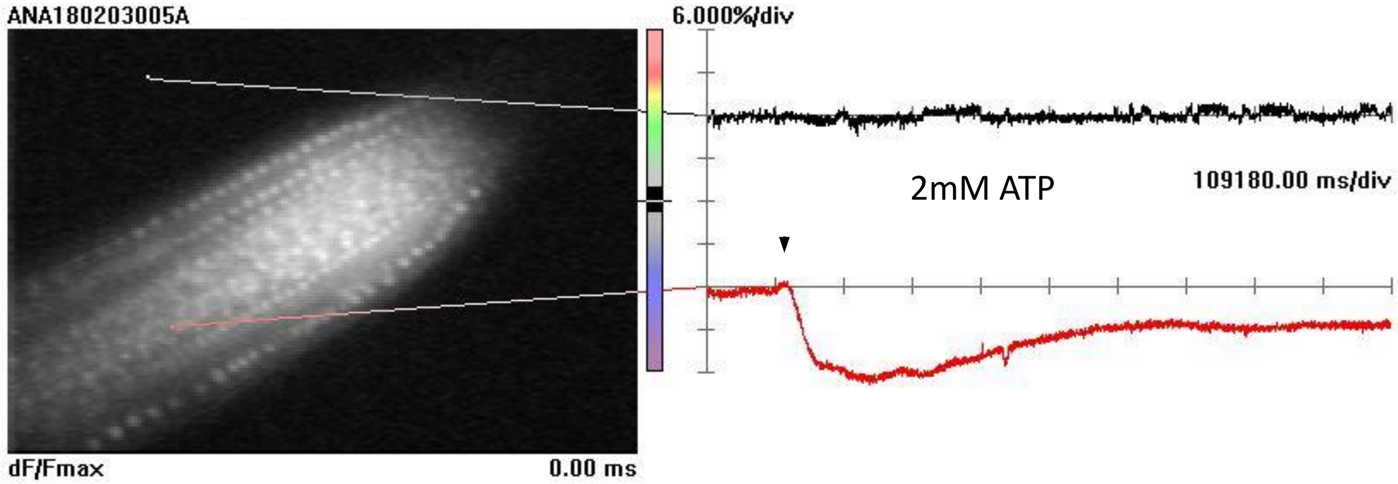

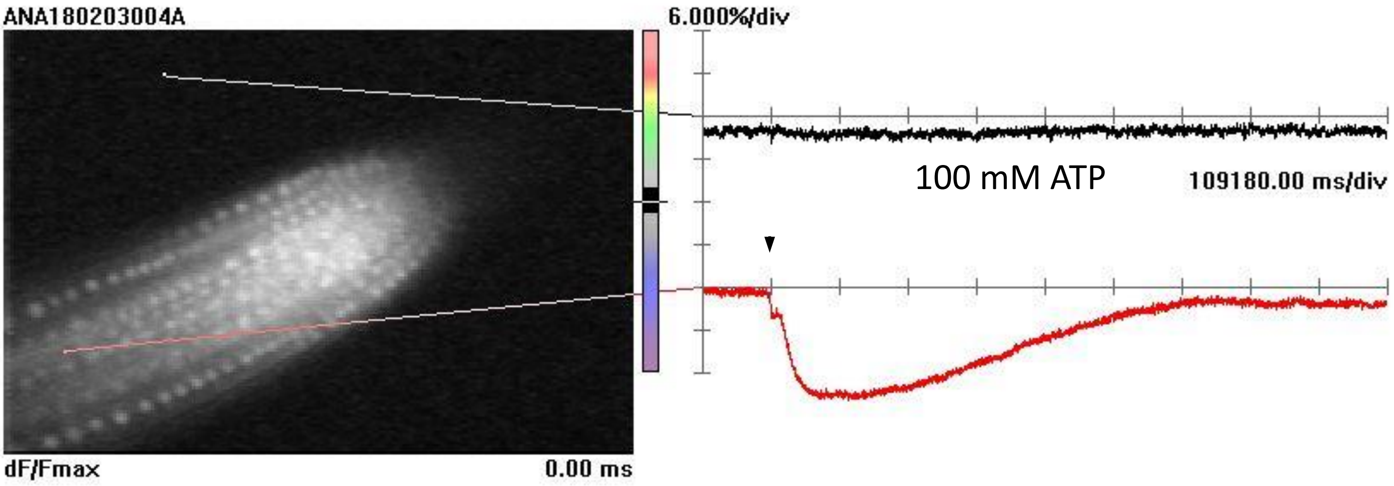

Data Sheet 2: Changes in membrane-bound and soluble SEpHluorinD fluorescence intensity in root tip cells in response to extracellular ATP (eATP). Representative data from one root n=1 are shown.

Experiments using SEpHluorinD targeted to the indicated locations indicate that application of eATP (arrowhead) provokes a pH change (reductions in SEpHluorinD fluorescence) over a period of 18 minutes at all sites tested. Method: Roots of living Arabidopsis seedlings were immobilized under an agarose block in a microscope slide dish (with the coverslip on the bottom of the dish). The root portions are then exposed to eATP by addition of eATP solutions (either 2 mM or 100 mM) to the edge of the agarose block. Buffer controls were also performed. Changes in SEpHluorinD fluorescence intensity in root tip cells were recorded using a high speed camera (MICAM02-HR, puchased from Major Instruments, Taiwan) mounted on an inverted microscope with a stable Xenon short ARC lamp (Ushio UXL-151DO from Ushio America) (Matzke and Matzke, 2015). The intensity changes were measured using BV-Analyzer(USB) x64 Edition Version 13. 12.20 from BrainVision Inc. Tokyo Japan.

Matzke, A.J.M., and Matzke, M. (2015). Expression and testing in plants of ArcLight, a genetically-encoded voltage indicator used in neuroscience research. BMC Plant Biol. 15, 245.

Figures shown on **pages 2-7** are magnifications of the six cellular locations (p. 1) shown for better viewing of the numbers:

These **Figures** show similar responses following either eATP or buffer addition of SEpHLuorinD targeted to different cellular locations: **(p. 2)** CBL1-SEpHluorinD [at the plasma membrane (PM)]; **(p. 3)** SEpHluorinD in the cytoplasm; **(p. 4)** WPP-SEpHluorinD [at the outer nuclear membrane (ONM) facing the cytoplasm]; **(p. 5)** SUN2-SEpHluorinD (at the inner nuclear membrane (INM) facing the perinuclear space (PNS); **(p. 6)** SEpHluorinD-SUN2 at the INM facing the nucleoplasm; and **(p. 7)** NLS3x- SEpHluorinD in the nucleoplasm.
The traces are derived from the regions of the root indicated by the lines connecting the traces (right) and the point of measurement in the image (left; MiCAM image, 20x objective) and are displayed over a time period of 1091800 milli-seconds (msec). Either buffer (top), 2 mM ATP (middle), or 100 mM ATP (bottom) was added at approximately 120000 msec as indicated by the black arrow-heads. Fractional fluorescence changes (%dF/Fmax) were calculated by the BV-Analyzer software supplied with the MiCAM camera. The divisions of the Y-axis are set at 6%. The X-axis shows time in msec (the devisions are set to 109180 msec) .
The indicated cells show a qualitatively response following addition of either eATP (2 mM and 100 mM) or buffer (red traces). The background trace, which remains unchanged following addition of eATP, is shown at the top of each trace in black.
