# Supplementary material for: Evidence That Ion-Based Signaling Initiating at the Cell Surface Can Potentially Influence Chromatin Dynamics and Chromatin-Bound Proteins in the Nucleus
Source: Front Plant Sci. 2019 Oct 17;10:1267. doi: 10.3389/fpls.2019.01267 (PMC6811650; doi:10.3389/fpls.2019.01267)
Supplement: Supplementary file 6 [file DataSheet_3.pdf]

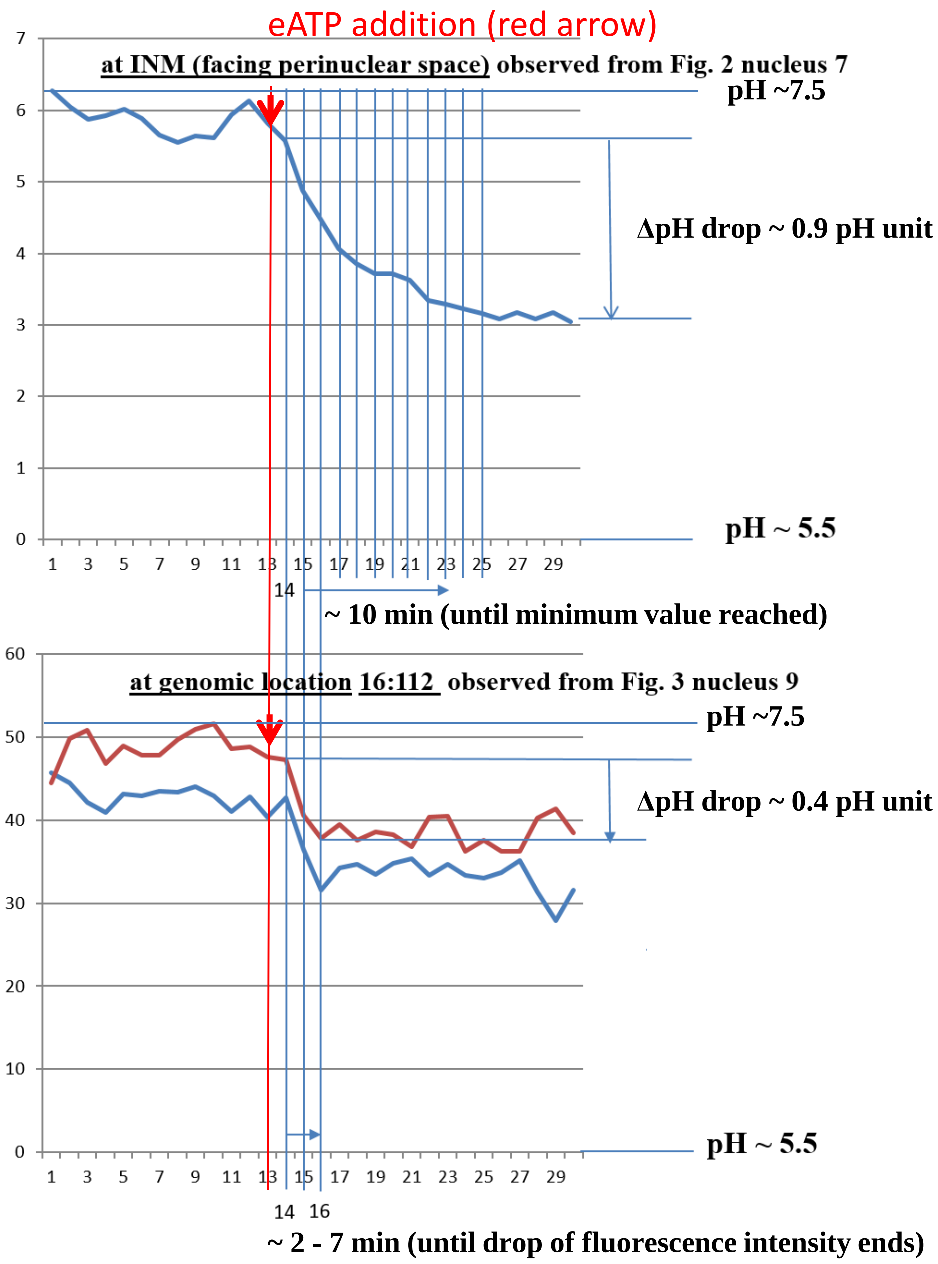

Data Sheet 3: Estimates of eATP-induced changes in pH at the INM (periplasmic face) and a genomic locus based on magnitude of reduction of SEpHluorin fluorescence

Left: The Y axis shows numbers returned by IMARIS representing fluorescence intensity. Right: the reported pH range of SEpHluorin fluorescence (maximum pH 7.5, extinguished pH 5.5; Miesenböck, 2012). The graphs depict representative results showing the observed drops in fluorescence and estimated pH changes at the INM (perinuclear space) and a genomic site over the time periods indicated after the addition of eATP. The corresponding figures and nucleus number are noted at the top of each graph.

Miesenböck, G. (2012). Genetically encoded reporters of synaptic transmission. Cold Spring Harb. Protoc. 2012, 213-217.

Data Sheet 3: Estimate of eATP-induced changes in pH at a genomic locus based on magnitude of reduction of SEpHluorin fluorescence

Looking at the series of graphs in Figures 3 and 5, it is important to differentiate between drops and continuous changes in fluorescence intensity. After adding eATP, we observe a drop in fluorescent intensity lasting ca. 2-7 min followed by leveling off. On top of that, we observe the continuous and irreversible decrease of fluorescence intensity because of bleaching. To be able to distinguish between drops of fluorescence intensity and continuous decreases, the experiments are carried out over a 30-minute time period. In the first 12 minutes, we can observe the amount of bleaching before eATP is added. At time-point 13 during the red channel acquisition, we add eATP and can then observe [or not observe in the case of mock (**Figure 3E**) or pH-insensitive-chromatin tag (**Figure 2E**, **Figure 4C**, and **Figure 5C right**)] a drop in fluorescence intensity lasting about 2-7 minutes until leveling off occurs. For the rest of the experiment, a continuous decrease in fluorescence intensity takes place because of bleaching.

Note that addition of eATP (or buffer) leads to transient ‘dislocation turbulence’ (up and/or down displacement of the root), which can result in a short flicker of fluorescent intensity change at time-point 13 in the red channel up and/or down or no change, [see **Figure 2E** (mRuby-LacR), and **Figure 5C right** (mRuby-LacR)]. In the green channel the ‘dislocation turbulence’ effect is much less pronounced [see **Figure 4C** with pH-insensitive chromatin tag (mCitrine-LacR)] because at time-point 14 the ‘dislocation turbulence’ caused by the addition of eATP during the red channel acquisition has already diminished, which allows us to be even more confident in the rapid changes observed in the green channel with pH sensitive chromatin tag (SEpHluorinD-LacR) (see **Figure 3D** , and **Figure 5C left**).
