# Supplementary material for: Evidence That Ion-Based Signaling Initiating at the Cell Surface Can Potentially Influence Chromatin Dynamics and Chromatin-Bound Proteins in the Nucleus
Source: Front Plant Sci. 2019 Oct 17;10:1267. doi: 10.3389/fpls.2019.01267 (PMC6811650; doi:10.3389/fpls.2019.01267)
Supplement: Supplementary file 7 [file DataSheet_4.pdf]

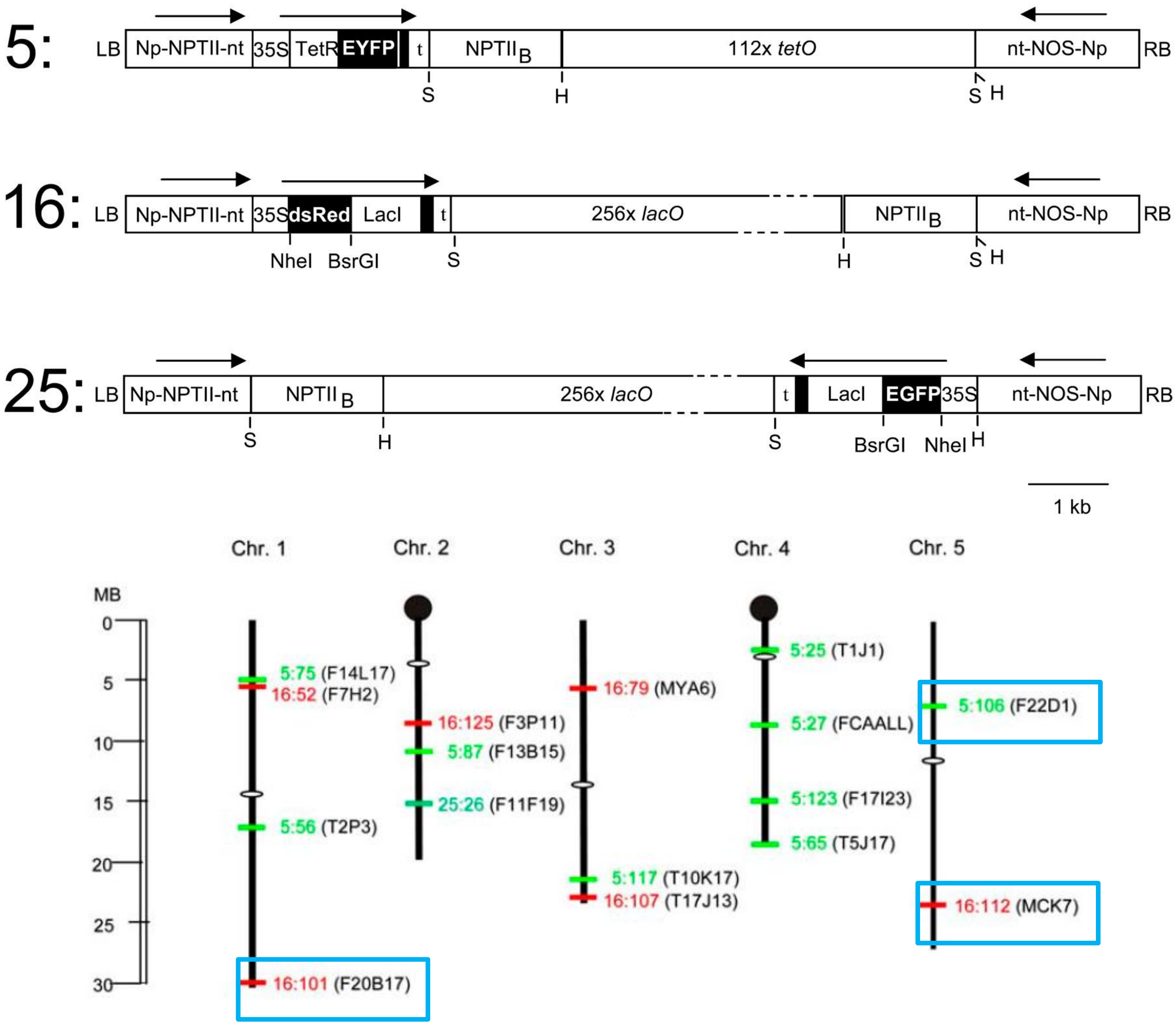

Data Sheet 4: Chromatin tagging constructs and positions of genomic insertion sites of operator repeats

Top: Constructs based on bacterial Tet and Lac operator-repressor systems used for fluorescent tagging of chromsomal sites. Each construct contains an operator repeat array (*tet* or *lac*) and a 35Spromoter-driven gene encoding a fusion protein comprising a fluoresence protein (FP)-repressor protein (RP) (TetR-EYFP, dsRed2-LacR, EGFP-LacR). For details see Matzke et al. (2005). Middle: Chromosomal locations in sixteen fluorescent-tagged lines using either the Tet system (EYFP; green) or the Lac system [dsRed2 (red) and EGFP (blue-green)] (Matzke et al., 2005). The boxed sites are used in the present study

Matzke, A.J.M., Huettel, B., van der Winden, J., and Matzke, M. (2005). Use of two-color fluorescence-tagged transgenes to study interphase chromosomes in living plants. Plant Physiol. 139, 1586-1596.
