# Supplementary material for: Evidence That Ion-Based Signaling Initiating at the Cell Surface Can Potentially Influence Chromatin Dynamics and Chromatin-Bound Proteins in the Nucleus
Source: Front Plant Sci. 2019 Oct 17;10:1267. doi: 10.3389/fpls.2019.01267 (PMC6811650; doi:10.3389/fpls.2019.01267)
Supplement: Supplementary file 8 [file DataSheet_5.pdf]

20µl Gilson
Pipetman with
Teflon tubing
1mm diameter

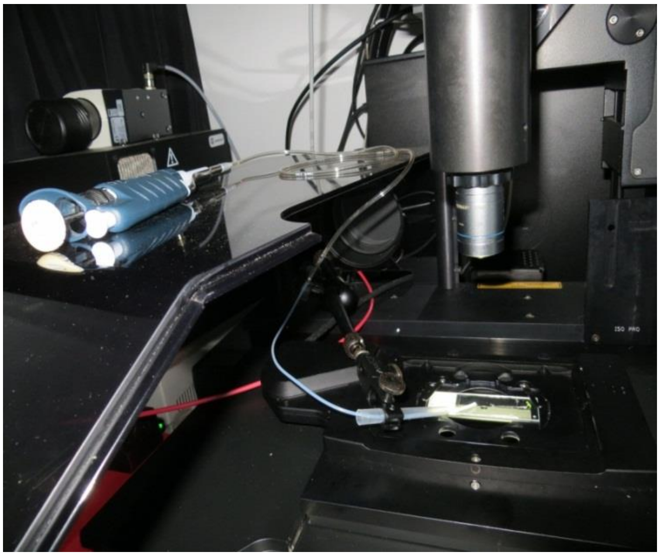

Teflon tubing
aimed at the
perforation
in the cover slip

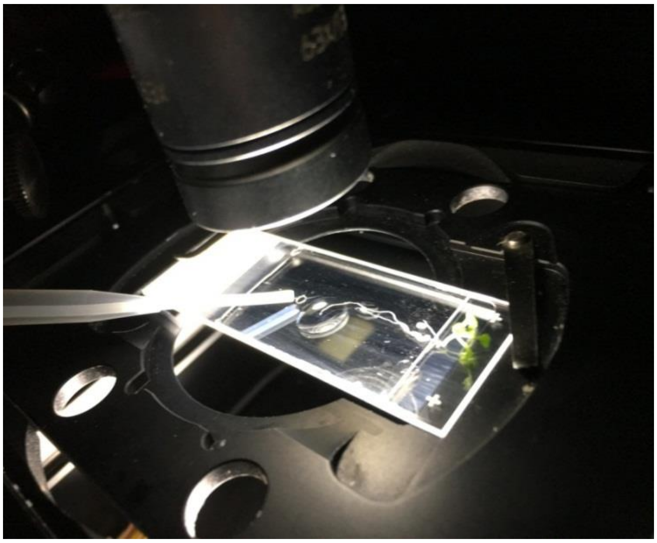

after lowering
63x objective

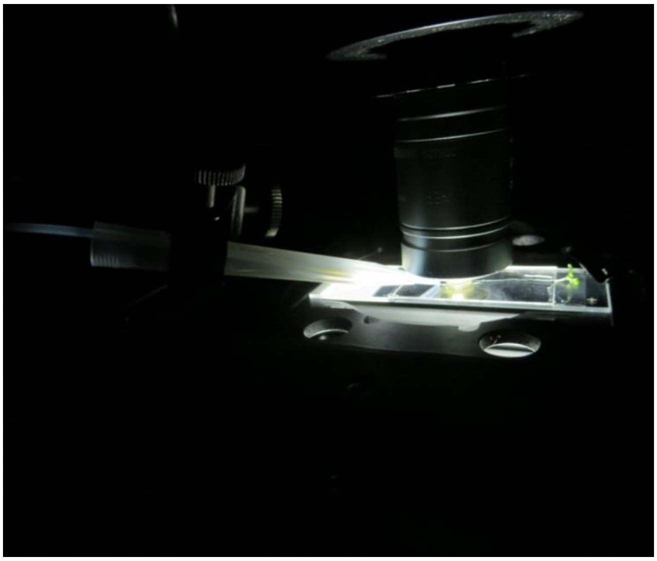

Data Sheet 5 Matzke et al

Data Sheet 5: Procedure for adding eATP during confocal microscopy and data acquisition

Top: View of the Gilson pipetman attached to teflon tubing held in place for aiming at the perforation in the cover slip
Middle: Close-up view of the teflon tubing over the perforation in the cover slip ~5 mm away from the root tip of the seedling mounted on a microscope slide
Bottom: View after lowering the 63X objective into place for starting data acquistion.
After 13 min during the red channel acquisition, the eATP is ejected from the pipetman through the perforation to reach the root tip. For further details, see Materials and Methods.
