# Supplementary material for: Evidence That Ion-Based Signaling Initiating at the Cell Surface Can Potentially Influence Chromatin Dynamics and Chromatin-Bound Proteins in the Nucleus
Source: Front Plant Sci. 2019 Oct 17;10:1267. doi: 10.3389/fpls.2019.01267 (PMC6811650; doi:10.3389/fpls.2019.01267)
Supplement: Supplementary file 9 [file DataSheet_6.pdf]

A)

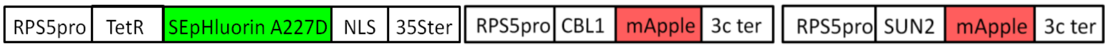

B)

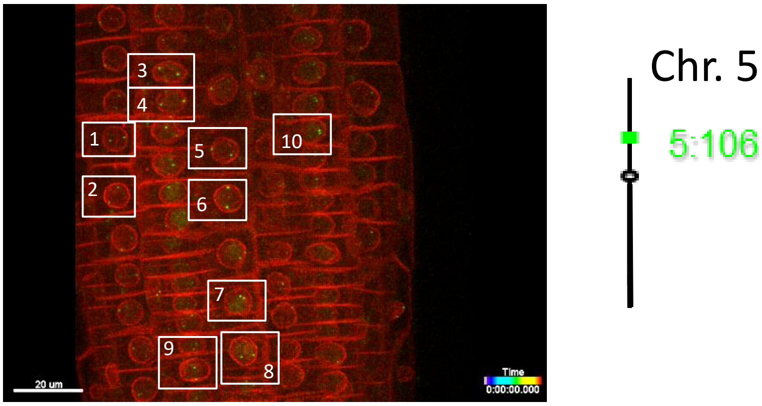

C) chromatin dynamics: -/+eATP

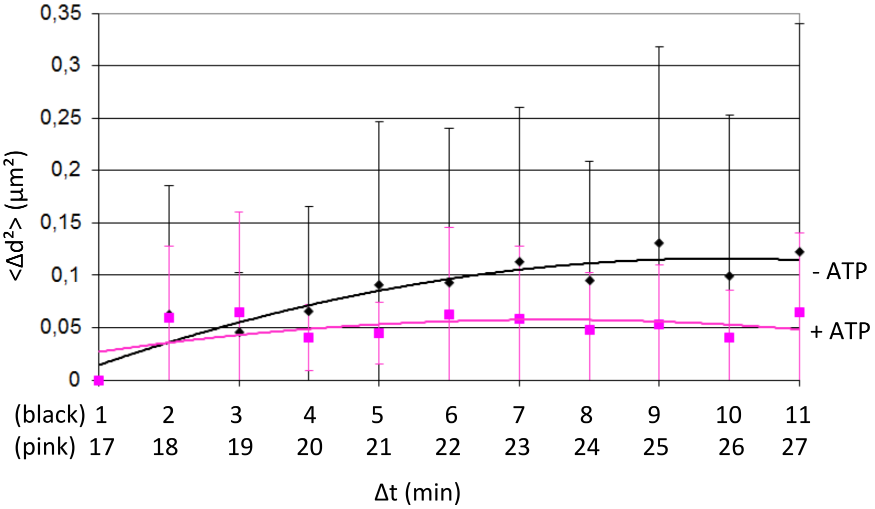

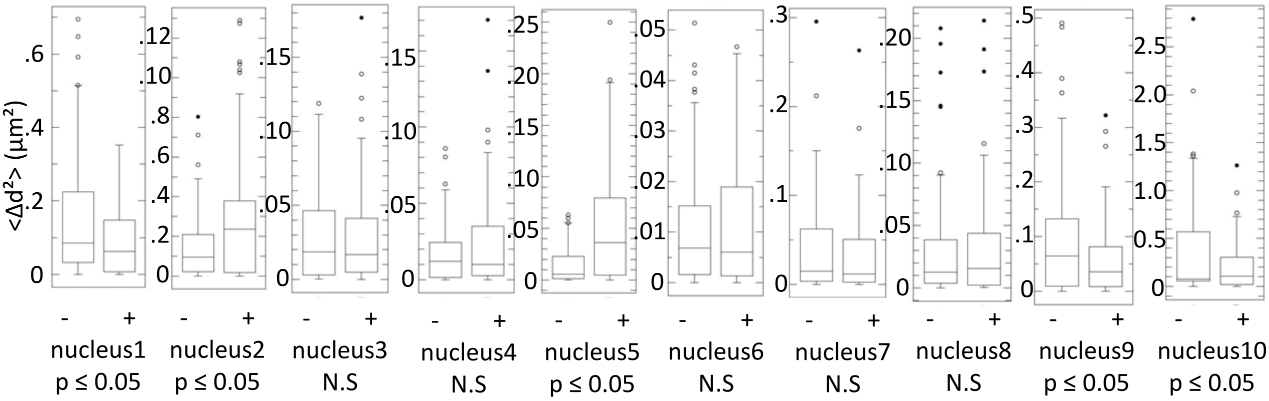

Data Sheet 6 Matzke et al

Data Sheet 6: Chromatin dynamics of locus 5:106 after addition of eATP

A) The construct encoded nuclear-localized TetR-SEpHluorinD fusion protein as a pH-sensitive fluorescent DNA binding protein, and SUN2-mApple and CBL1-mApple as INM and PM visual markers. All three genes are under the transcriptional control of the RPS5 promoter; the first contains the 35ter and the last two contain the 3C terminator (3Cter) **(Data Sheet 1, combination Data Sheet 6).**

B) Confocal image (maximum projection) at time point t1 of homozygous fluorescent-tagged locus 5:106 in nuclei of cells in the root transition zone (an enlarged confocal image is shown in **Data Sheet 9**). The TetR-SEpHluorinD fusion protein binds to *tetO* repeats integrated at locus 5:106 on the bottom arm of chromosome 5. Two green dots are visible in most nuclei. mApple, which bleaches rapidly after time point t1, is used only for defining the INM and PM. Nuclei used for chromatin dynamics analysis (part C) are boxed in white. Mounting, confocal microscopy, eATP treatment, data acquisition and data analysis of *Arabidopsis* seedlings harboring the above construct were carried out as described in the legend to **Figure 2**.

C) Chromatin dynamics was determined as described in the legend to **Figure 1**. **Top:** The graph shows two lines, which indicate 'jiggling' before (black) and after (pink) eATP addition**.** Time points 12-16 were excluded from the analysis owing to data during dislocation turbulence caused by addition of eATP. To detect differences in the radius of confinement before and after eATP treatment, the analysis was restarted following the addition of eATP (following cessation of dislocation turbulence), hence producing two lines (black and pink) (**Table 2, sheet7).** Standard deviation bars at each time point are shown (calculated from data in **Table 7). Bottom:** Single nuclei box plot analyses and calculation of p values using the Δd^2 square values revealed three nuclei with significant changes ($p \leq 0.05$) in the mobility of locus 5:106 following eATP treatment. N.S., not significant
