# Supplementary material for: Evidence That Ion-Based Signaling Initiating at the Cell Surface Can Potentially Influence Chromatin Dynamics and Chromatin-Bound Proteins in the Nucleus
Source: Front Plant Sci. 2019 Oct 17;10:1267. doi: 10.3389/fpls.2019.01267 (PMC6811650; doi:10.3389/fpls.2019.01267)
Supplement: Supplementary file 10 [file DataSheet_7.pdf]

(1) allelic distances

1 2 3 4 5 6 7 8 9 10

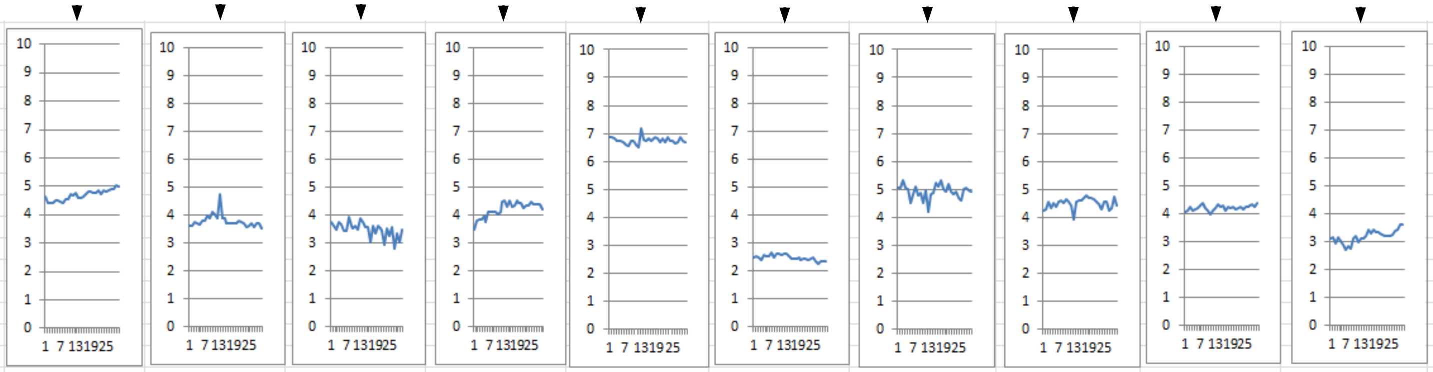

(2) intensity of nuclei: #s on bottom are estimated Δ pH values upon eATP addition

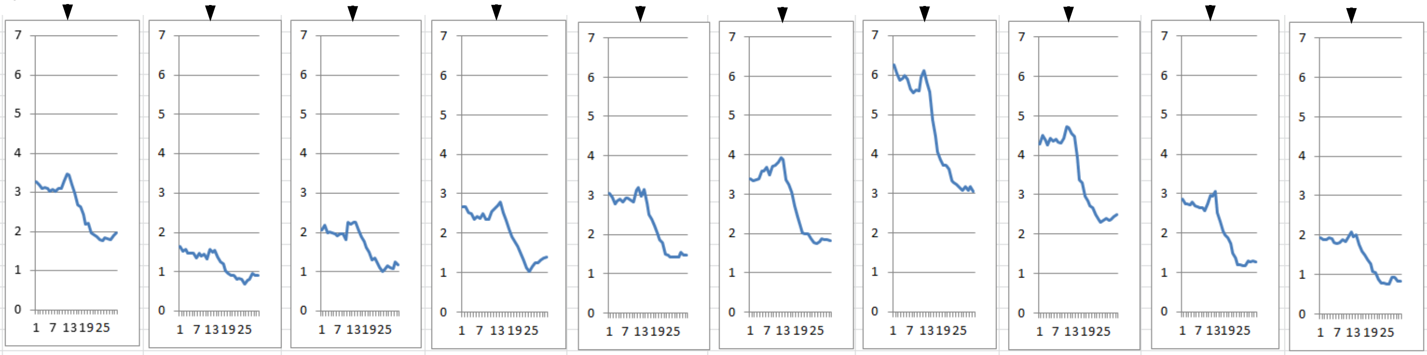

0.5 0.3 0.4 0.5 0.5 0.7 0.9 0.7 0.6 0.4

(3) nuclear volumes

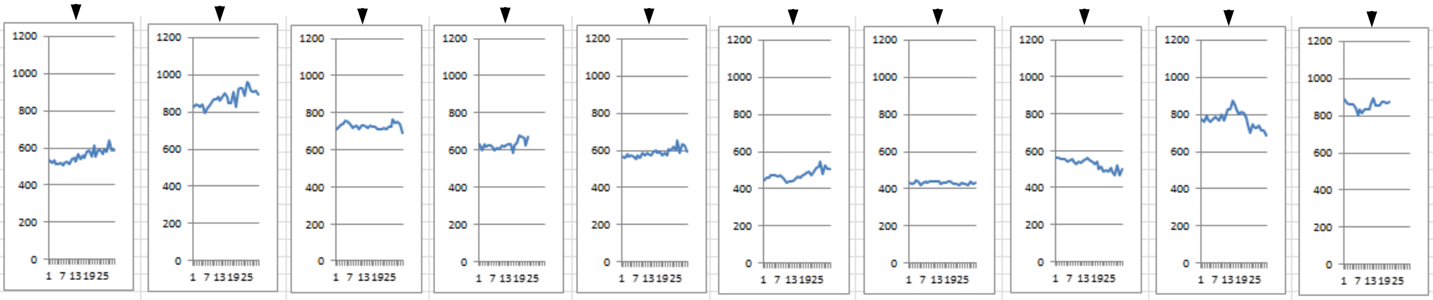

(4) distances of alleles from INM

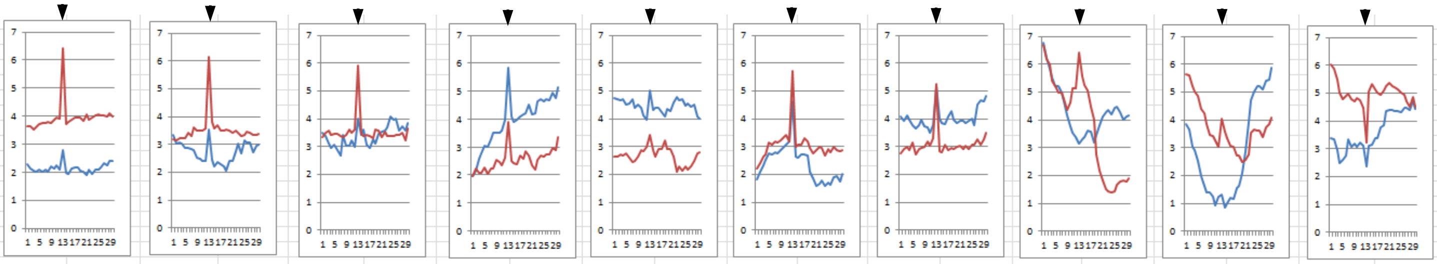

Data Sheet 7 Matzke et al

Data Sheet 7: Consequence of eATP addition for four observed parameters

Estimates of (1) allelic distances, (2) ΔpH at the INM, (3) nuclear volumes, and (4) distances of alleles from the INM in the ten nuclei over the time of the experiment described in **Figure 2** are shown.

The four graphs compare: 1. **allelic distance** data of nuclei 1-10 of **Figure 2** are shown at all the timepoints of the experiment; **2. fluorescent intensity data of INM**, with estimated Δ **pH** values after eATP addition at the bottom of the graphs; **3. nuclear volumes; 4**. **distances of all alleles from the INM** at all timepoints of the experiment. Arrowheads above the graphs indicate the time-point of eATP addition. The numbers on the Y axes are returned by Imaris.

The results show that after addition of eATP, changes can occur in all 4 observed parameters :

1. **Allelic distance changes** after eATP addition can become more or less frequent and stronger or weaker in amplitude. Raw data in **Table 2, sheet 5**.
2. Δ **pH** values after eATP addition vary among nuclei between 0.3 to 0.9 in this experiment. Δ **pH** values determined as shown in Supplementary Figure S3. Raw data (fluorescent intensity values from Imaris are found in **Table 1, sheet 1)**.
3. **Nuclear volume** measurements were retrieved from the 3D image time-laps data. The nuclear volume does not show consistent changes to possible thermic (illumination)-induced swelling, indicating that such swelling does not occur. Nuclear volumes can shrink (nucleus 8,9), be stable (nucleus 3 and 7) or show some swelling. Thus, it can be ruled out that there is thermic(illumination)-induced swelling of nuclei (which would then in turn affect the spreading of chromosomes, causing slight chromosomal dispersion) during the time of the experiment. Raw nuclear volume data of nuclei in Figure 2 are shown in **Table 9.**
4. **Distances of all alleles from the INM** were found to be very variable and independent of each other. After eATP addition, similar or opposite directional movments (either moving closer to INM or moving further away from INM) were observed in this experiment. In a given nucleus, one allele is shown in red, the other in blue. The large spikes at time-point 13 indicate dislocation turbulence upon addition of eATP. Raw data of distances of alleles to the INM in Figure 2 are shown in **Table 8**.
