# Supplementary material for: Evidence That Ion-Based Signaling Initiating at the Cell Surface Can Potentially Influence Chromatin Dynamics and Chromatin-Bound Proteins in the Nucleus
Source: Front Plant Sci. 2019 Oct 17;10:1267. doi: 10.3389/fpls.2019.01267 (PMC6811650; doi:10.3389/fpls.2019.01267)
Supplement: Supplementary file 11 [file DataSheet_8.pdf]

Figure 2D left (top), 2E right (bottom)

pH-sensitive INM (SUN2-SEpHluorinD): fluorescence intensity changes +eATP

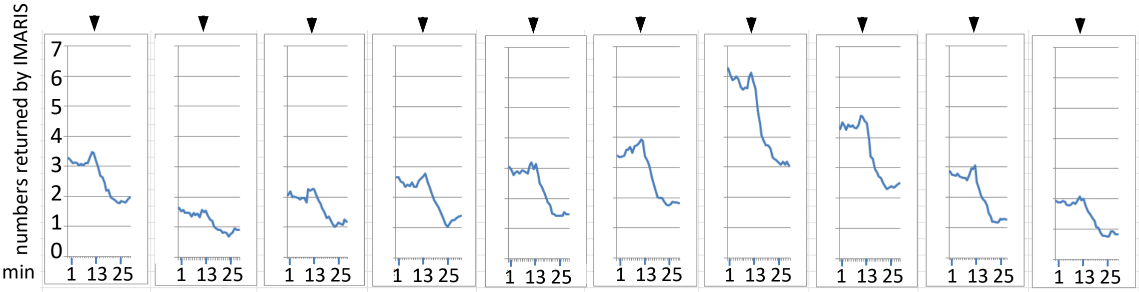

nucleus	1	2	3	4	5	6	7	8	9	10
Δ pH	0.5	0.3	0.4	0.5	0.5	0,7	0.9	0.7	0.6	0.4

pH-insensitive chromatin tag (mRuby-LacR): bleaching (and dislocation turbulance) +eATP (2 alleles)

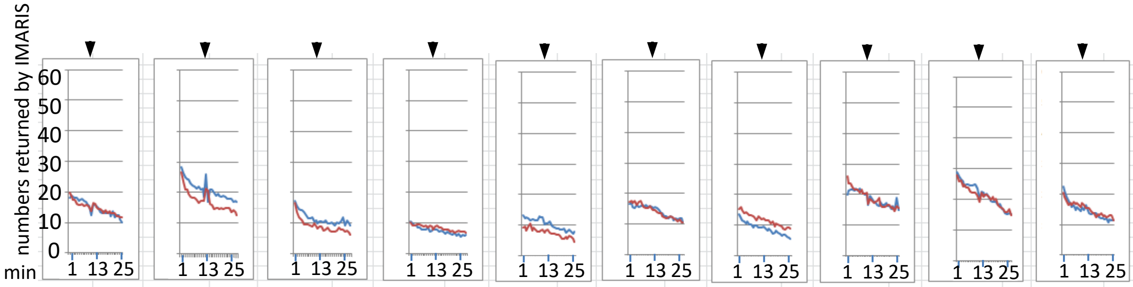

nucleus	1	2	3	4	5	6	7	8	9	10

Figure 3D left (top), 3E right (bottom)

pH-sensitive chromatin tag (SE-pHluorinD): fluorescence intensity changes +eATP (2 alleles)

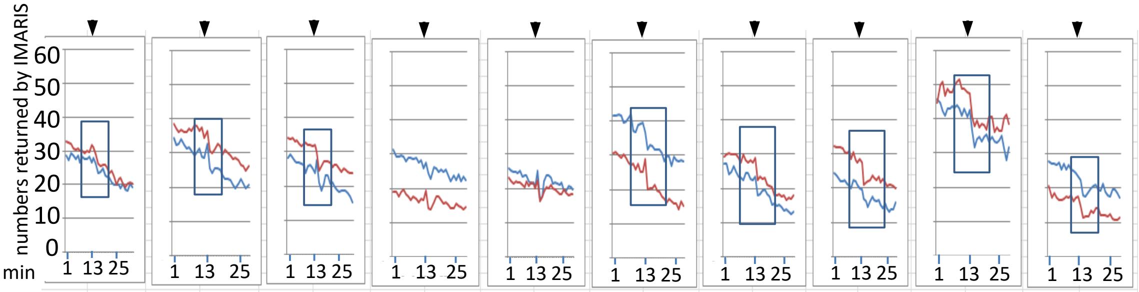

nucleus	1	2	3	4	5	6	7	8	9	10
Δ pH allele1	0.2	0.2	0,2	0.1	0.1	0.3	0.2	0.2	0.4	0.2
Δ pH allele2	0.2	0.2	0.2	0.1	0.1	0.2	0.2	0.3	0.4	0.2

buffer control: bleaching (and dislocation turbulance) + buffer (2 alleles)

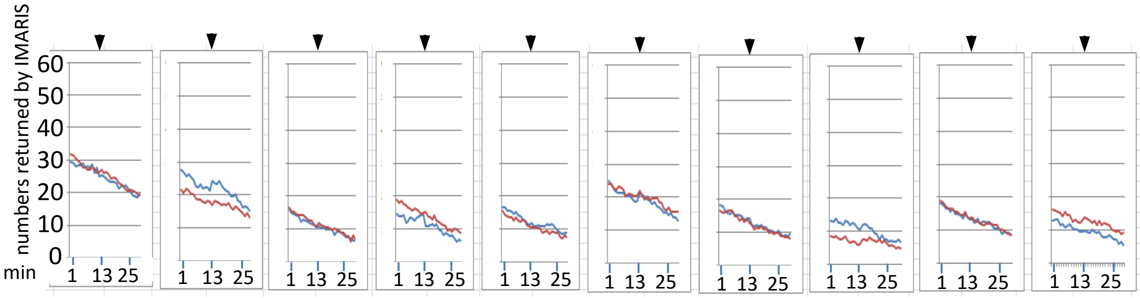

nucleus	1	2	3	4	5	6	7	8	9	10

Figure 4C

pH-insensitive chromatin tag (mCitrine-LacR): bleaching (and dislocation turbulance) +eATP (2 alleles)

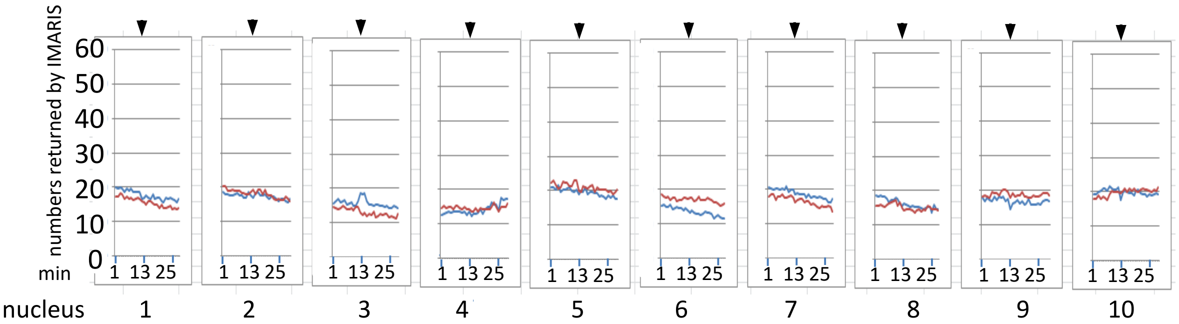

Figure 5C_left (top), 5C_right (bottom)

fluorescence intensity changes of dual-colored alleles

pH-sensitive chromatin tag (SEpHluorinD-LacR): fluorescent intensity changes +eATP (2 alleles)

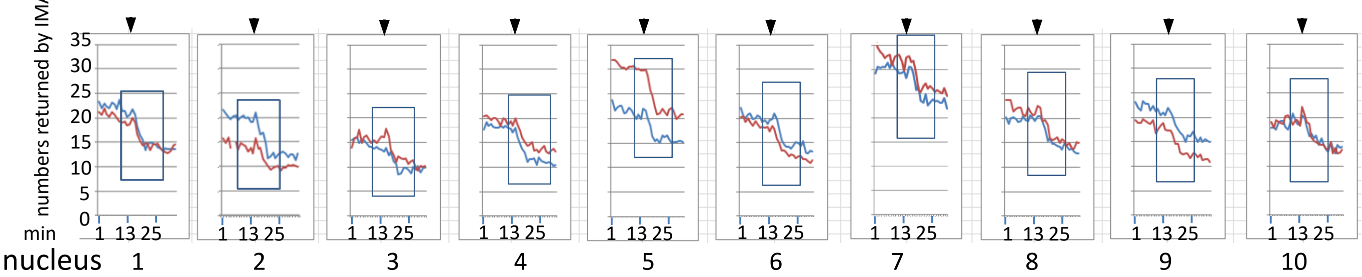

	1	2	3	4	5	6	7	8	9	10
Δ pH allele1	0.4	0.4	0,3	0.3	0.3	0.3	0.3	0.3	0.2	0.2
Δ pH allele2	0.2	0.2	0.3	0.2	0.5	0.2	0.3	0.3	0.3	0.2

pH-insensitive chromatin tag (mRuby–LacR): bleaching (and dislocation turbulence) +eATP (2 alleles)

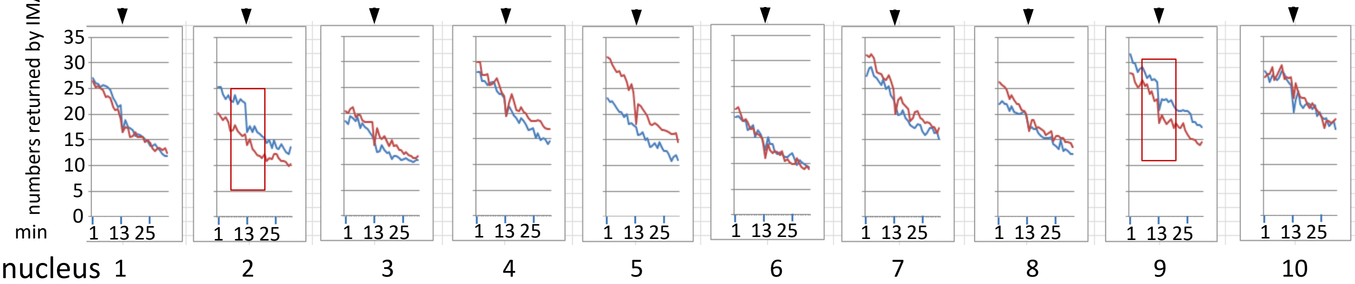

Data Sheet 8 Matzke et al

Data Sheet 8. Fluorescence intensities in individual nuclei

Figure 2D left (top)
Top: Fluorescence intensity values (returned by Imaris) of INM-localized SEpHluorinD (numbers can be viewed in **Table 1, sheet 1)** after addition of eATP (arrowhead) in ten white-boxed nuclei shown in Figure2 part B, ΔpH values are shown under each nucleus1-10 (maximum ΔpH 0.9, minimum ΔpH 0.3; average (n=10) ΔpH 0.6). Fluorescence intensity at INM is read in spot objects (spot size ca. 10 µm) capturing punctual fluorescence.

Figure 2E right (bottom)
Bottom: Response of the pH-insensitive mRuby-LacR chromatin tag following eATP treatment (numbers can be viewed in **Table 1, sheet 2)**. Fluorescence intensities (values returned by IMARIS) of two alleles of locus 16:101 (red and blue lines respectively) in ten white boxed nuclei (numbered in black below the graphs and in white in Figure2 part B). The fluorescence intensity at the genomic location is read in spots objects (1-2.8 µm) capturing punctual fluorescence of the tagged regions.

Figure 3D left (top)
Top: Fluorescence intensity profiles (values returned by Imaris) of the two 16:112 alleles (red and blue lines, respectively) in the ten white-boxed nuclei (numbered in black below the graphs and in white in Figure3 part B) following eATP addition (black arrowheads at frames 13-14). Blue boxed areas in the fluorescent intensity graphs highlight the region of interest (see text). ΔpH values are shown under each nucleus1-10 for both alleles [maximum ΔpH 0.4 (nucleus 9); minimum ΔpH 0.1 (nuclei 4 and 5); average ΔpH 0.2 (n=20)] (numbers shown in **Table 1, sheet 3)**.

Figure 3E right (bottom)
Bottom: Buffer control (without eATP) (numbers shown in **Table 1, sheet 4)**. The spikes in fluorescence reflect dislocation turbulence, since they are observed upon addition of eATP or buffer. The fluorescence intensity at the genomic location is read in spots objects (1-2.8 µm) capturing punctual fluorescence of the tagged regions.

Figure4C

Fluorescence intensity profiles (values returned by Imaris) of the two 16:101 alleles (red and blue lines, respectively) in the ten white-boxed nuclei (numbered in black below the graphs and in white in Figure4 part B) following eATP addition (black arrowheads at frames 13-14) (numbers shown in **Table 1, sheet 5).** Fluorescence intensity at genomic location is read in spot objects (1-2.8 µm) capturing punctual fluorescence.

Figure 5C left (top)

Top: Fluorescence intensity profiles (values returned by Imaris) of the two 16:112 alleles of the pH-sensitive tag (red and blue lines, respectively) in ten white-boxed nuclei (numbered below the graphs in black and in white in Figure5 part C) following eATP addition (black arrowheads at frame 13-14). ΔpH values are shown under each nucleus1-10 (maximum ΔpH 0.5 (nucleus5 second allele), minimum ΔpH 0.2 (nuclei 1,2,4,6,9,second allele only, 10 both alleles) average ΔpH 0.3 (n=20)).

Figure 5C right (bottom)

Bottom: response of pH-insensitive mRuby-LacR to eATP. The spikes in fluorescence reflect dislocation turbulence following eATP application (numbers can be viewed in **Table 1, sheet 6).** Fluorescence intensity at genomic location is read in spot objects (1-2.8 µm) capturing punctual fluorescence. The immediate reductions in fluorescence in nuclei 2 and 9 (**bottom**, red boxes), which affect primarily a single allele, are presumably due to a technical artefact.
