# Supplementary material for: Evidence That Ion-Based Signaling Initiating at the Cell Surface Can Potentially Influence Chromatin Dynamics and Chromatin-Bound Proteins in the Nucleus
Source: Front Plant Sci. 2019 Oct 17;10:1267. doi: 10.3389/fpls.2019.01267 (PMC6811650; doi:10.3389/fpls.2019.01267)
Supplement: Supplementary file 12 [file DataSheet_9.pdf]

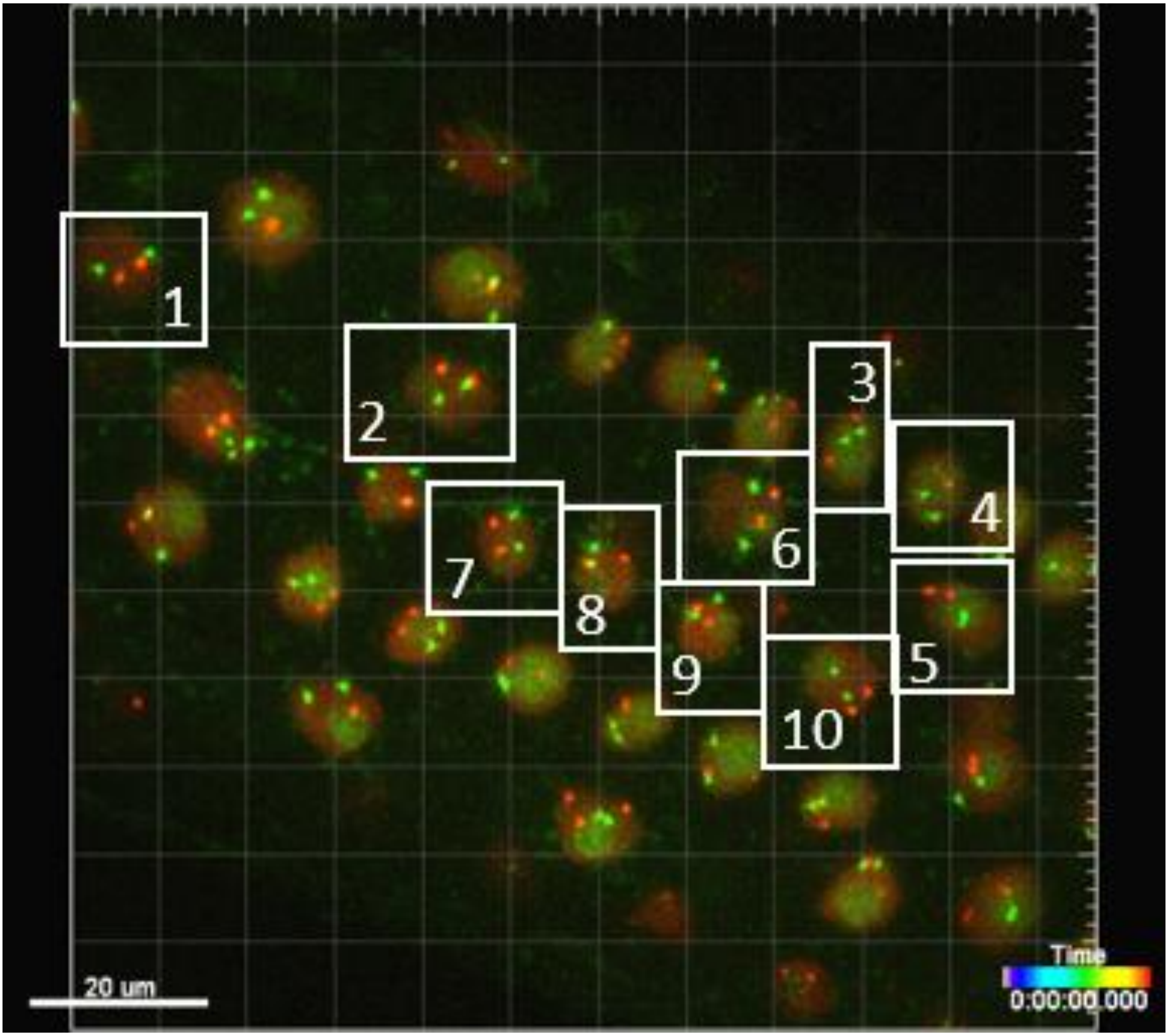

Figure 1B top (enlarged)

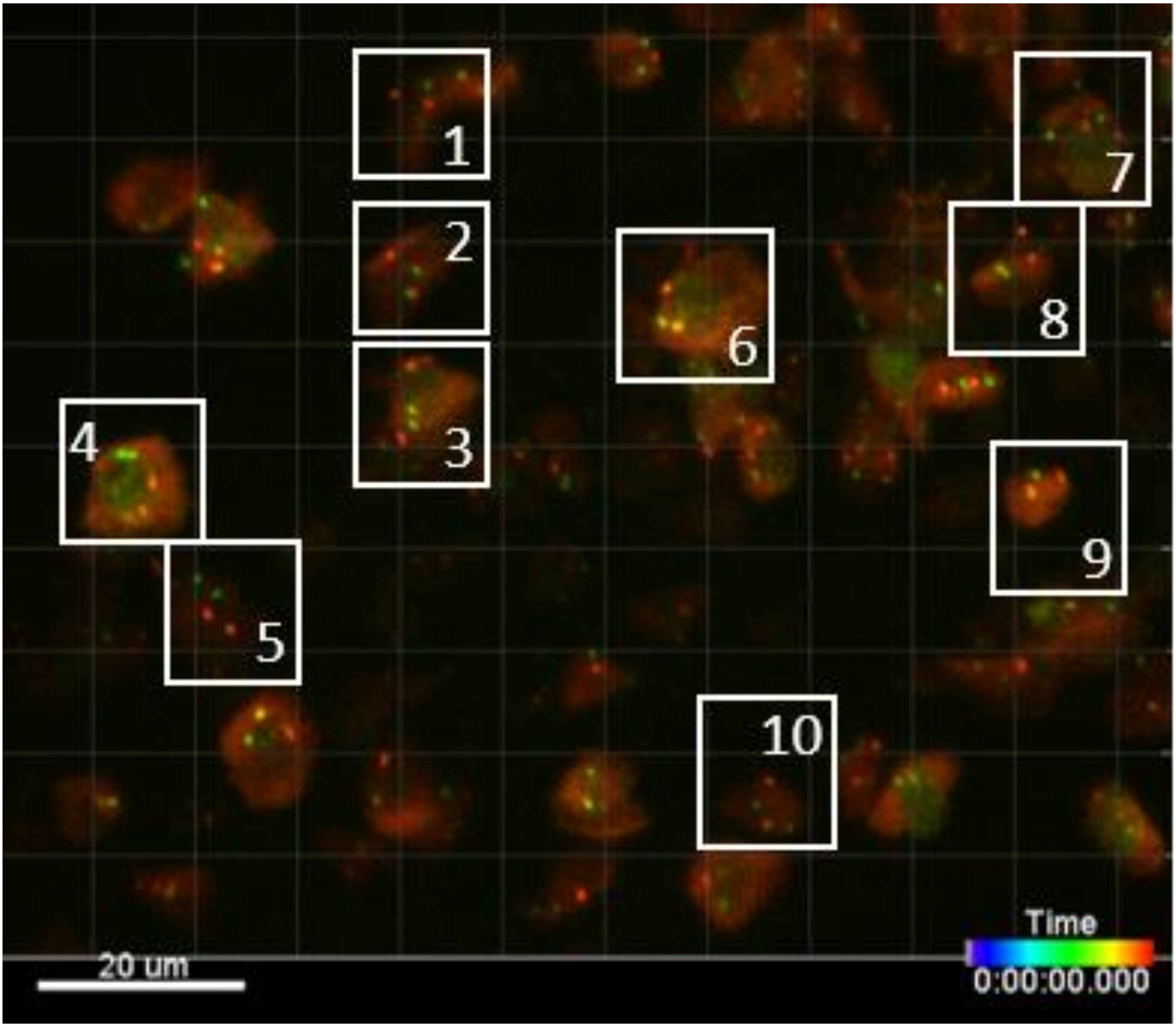

Figure 1D bottom (enlarged)

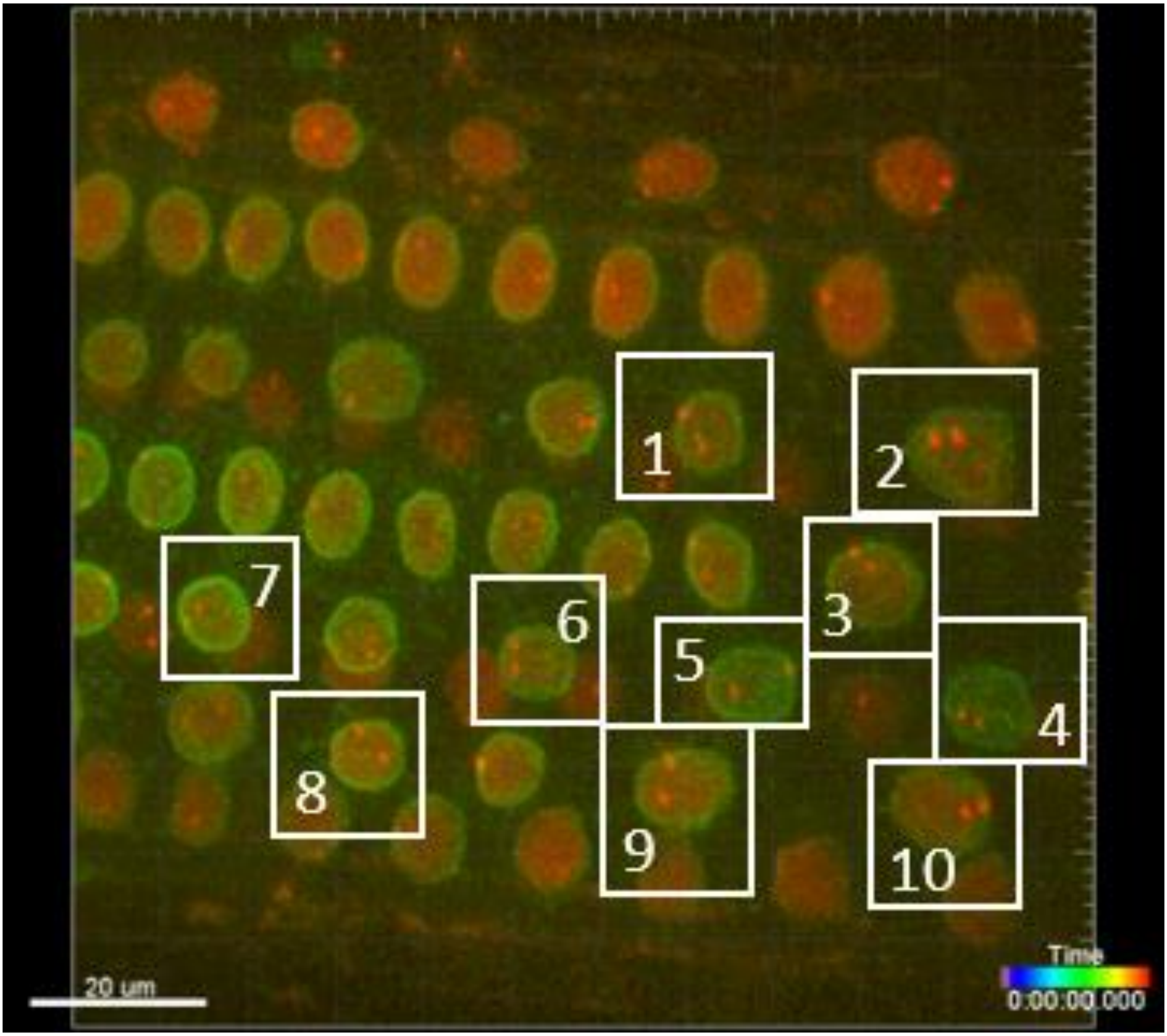

Figure 2B
(enlarged)

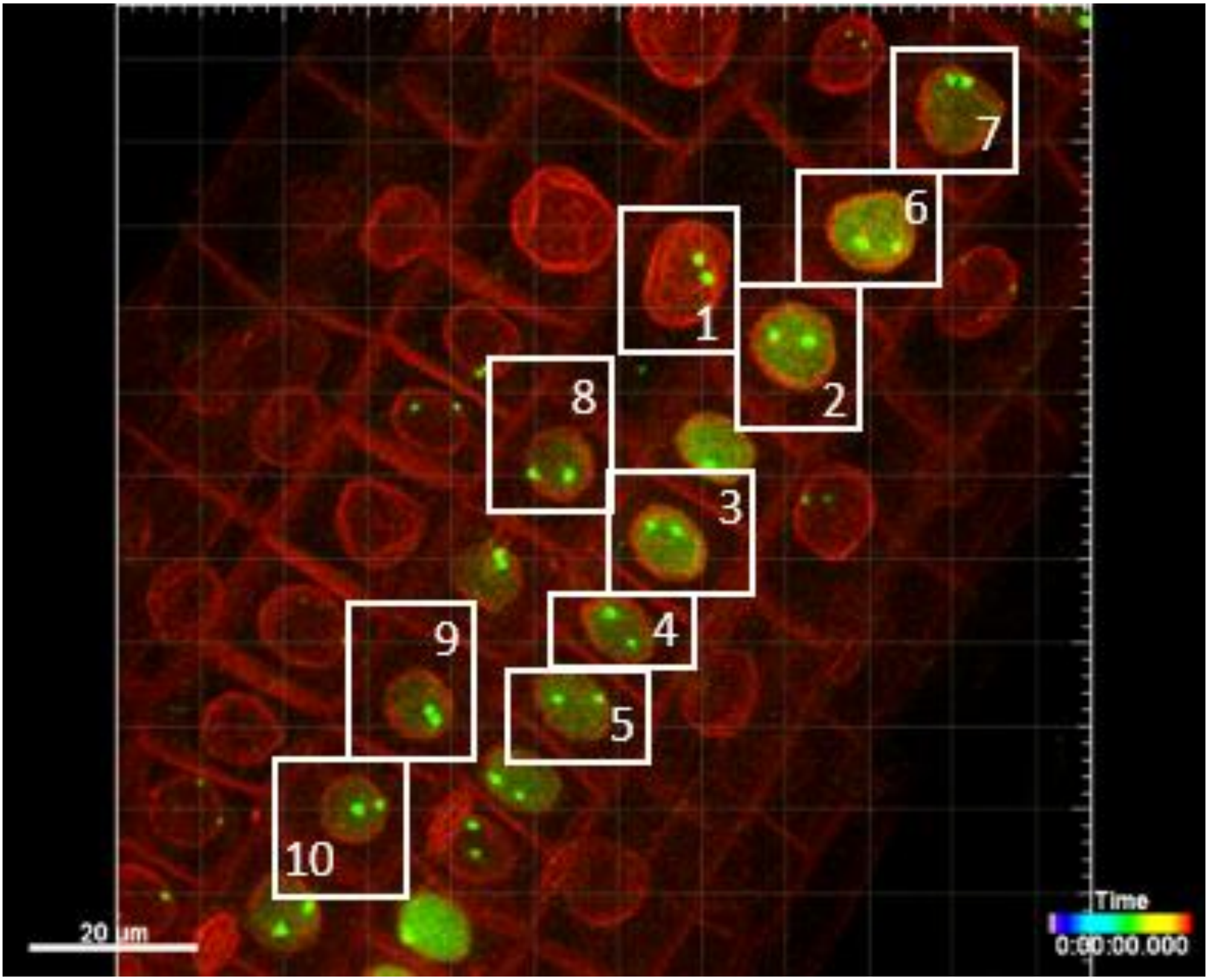

Figure 3B top (enlarged)

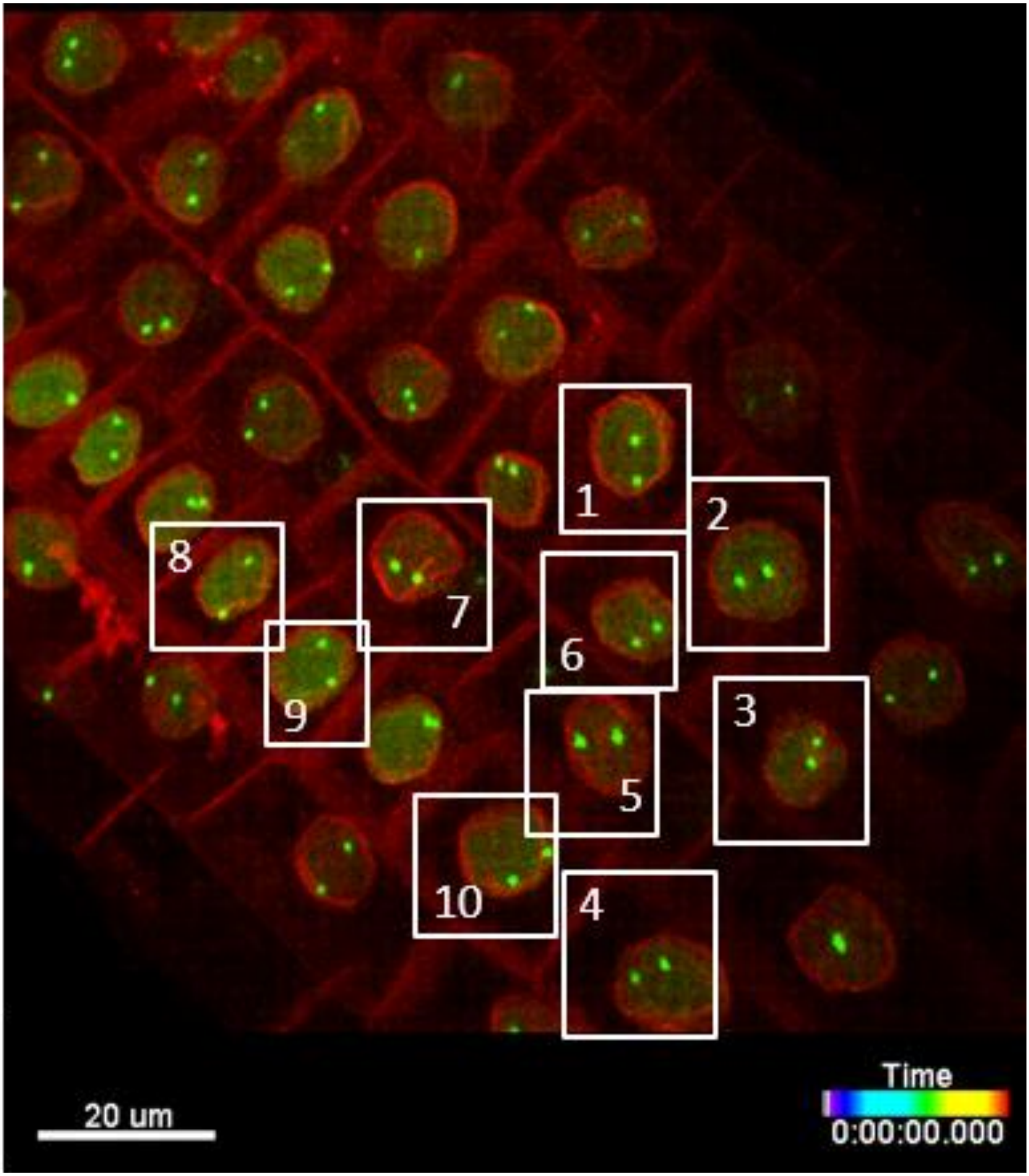

Figure 4B
(enlarged)

Data Sheet 9 Matzke et al

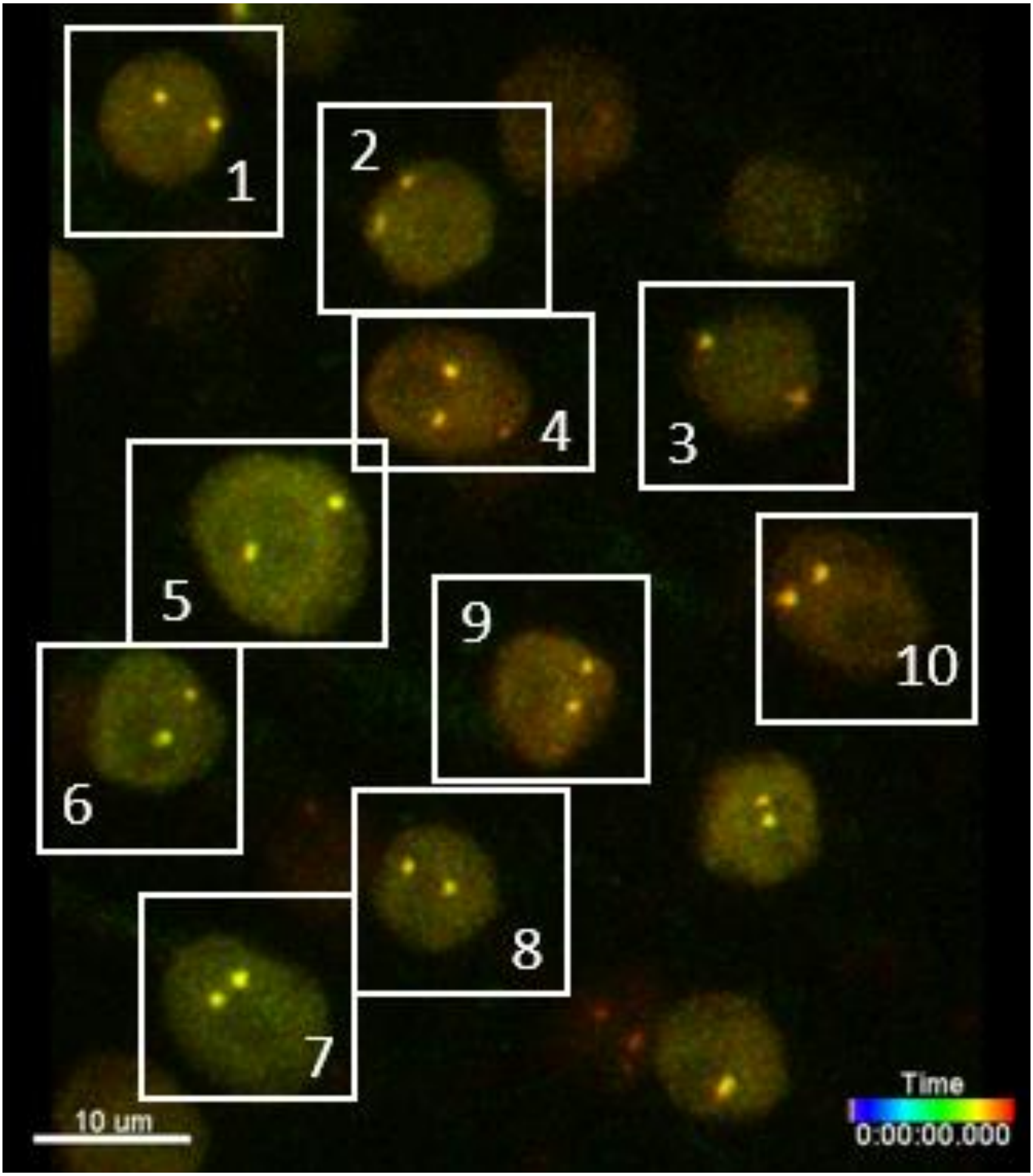

Figure 5B (enlarged)

Data Sheet 9 Matzke et al

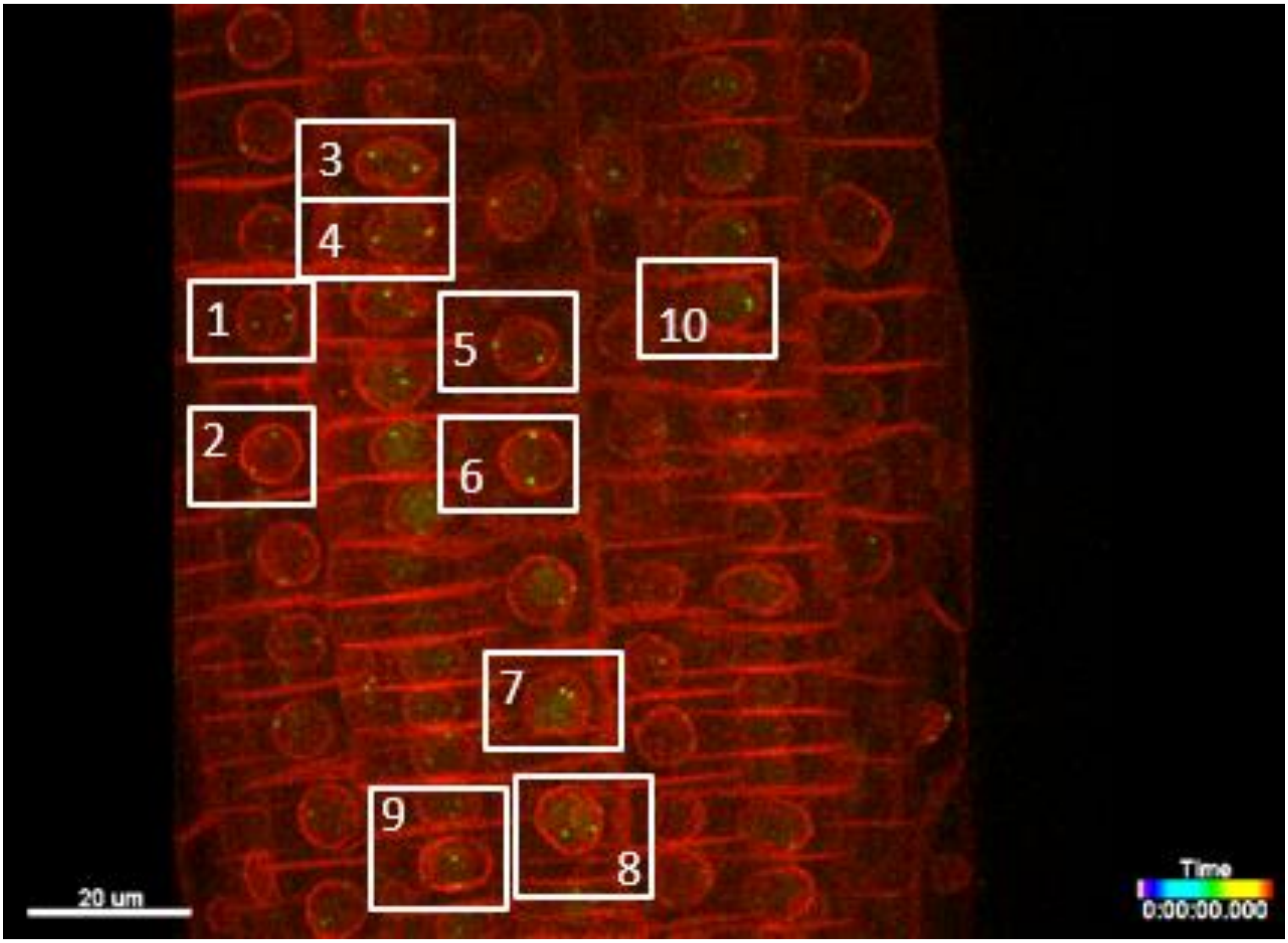

Data Sheet 6B (enlarged)
